# Optimising Clinical Epidemiology in Disease Outbreaks: Analysis of ISARIC-WHO COVID-19 Case Report Form Utilisation

**DOI:** 10.3390/epidemiologia5030039

**Published:** 2024-08-30

**Authors:** Laura Merson, Sara Duque, Esteban Garcia-Gallo, Trokon Omarley Yeabah, Jamie Rylance, Janet Diaz, Antoine Flahault, ISARIC Clinical Characterisation Group

**Affiliations:** 1 ISARIC, Pandemic Sciences Institute, University of Oxford, Oxford OX37LF, UK; sara.duquevallejo@ndm.ox.ac.uk (S.D.); esteban.garcia@ndm.ox.ac.uk (E.G.-G.); 2 Institute of Global Health, Faculty of Medicine, University of Geneva, 1202 Geneva, Switzerland; antoine.flahault@unige.ch; 3 Infectious Diseases Department, Universidad de La Sabana, Chia 250001, Colombia; 4 National Public Health Institute, Monrovia 1000, Liberia; trokonyeabah@gmail.com; 5 Health Emergencies Program, World Health Organization, 1211 Geneva, Switzerland; rylancej@who.int (J.R.); diazj@who.int (J.D.)

**Keywords:** clinical epidemiology, infectious disease outbreaks, data collection, data management, common data elements, ISARIC

## Abstract

Standardised forms for capturing clinical data promote consistency in data collection and analysis across research sites, enabling faster, higher-quality evidence generation. ISARIC and the World Health Organization have developed case report forms (CRFs) for the clinical characterisation of several infectious disease outbreaks. To improve the design and quality of future forms, we analysed the inclusion and completion rates of the 243 fields on the ISARIC-WHO COVID-19 CRF. Data from 42 diverse collaborations, covering 1886 hospitals and 950,064 patients, were analysed. A mean of 129.6 fields (53%) were included in the adapted CRFs implemented across the sites. Consistent patterns of field inclusion and completion aligned with globally recognised research priorities in outbreaks of novel infectious diseases. Outcome status was the most highly included (95.2%) and completed (89.8%) field, followed by admission demographics (79.1% and 91.6%), comorbidities (77.9% and 79.0%), signs and symptoms (68.9% and 78.4%), and vitals (70.3% and 69.1%). Mean field completion was higher in severe patients (70.2%) than in all patients (61.6%). The results reveal how clinical characterisation CRFs can be streamlined to reduce data collection time, including the modularisation of CRFs, to offer a choice of data volume collection and the separation of critical care interventions. This data-driven approach to designing CRFs enhances the efficiency of data collection to inform patient care and public health response.

## 1. Introduction

Clinical data are the foundation of an evidence-based response to outbreaks—at both the clinical and policy levels. The case definition, time to symptom progression, rates and covariates of clinical outcomes, and risk factors for severe disease or fatality must be rapidly defined to manage novel infections or unexpected changes in the epidemiology of known infections [1]. However, the collection of clinical data relies on the availability and time of front-line health care workers and research staff. This is especially true in low-resource settings where electronic health records are less common and disease outbreaks are more common [2,3]. As health emergencies often cause an increase in clinical caseload and other disruptions to health systems [4,5], it is important to minimise the burden of data collection to avoid detracting from patient care responsibilities. Evidence and innovations are required to design more efficient data tools that continue to address key knowledge gaps while optimising the use of limited staff resources.

For more than a decade, ISARIC (the International Severe Acute Respiratory and emerging Infections Consortium) and the World Health Organization (WHO) have developed case report forms (CRFs) that collect data to characterise the key clinical features of emerging infectious diseases [6]. These forms have been used in response to outbreaks of MERS-CoV, Ebola virus disease, Zika, severe acute respiratory illness, acute non-A-E hepatitis, and Mpox [7,8,9,10,11,12]. In January 2020, upon recognition of the need for urgent research on the novel SARS-CoV-2, ISARIC and WHO adapted their existing data forms and created a paper and electronic CRF to characterise COVID-19 [13]. The form was used by thousands of sites around the world to collect data that informed local, regional, and international pandemic responses [14]. While some sites used the form in its original design [15,16,17,18], others used it as a template to create a locally tailored version [19,20,21]. Requests for support to amend the forms for specific contexts, including low-resource settings and critical care units, resulted in the creation of an ISARIC-WHO ‘Rapid’ COVID-19 CRF and add-on modules with bespoke fields (or questions) for special populations, e.g., pregnant women. All forms and modules were openly accessible, available in six languages. Data management support was provided by ISARIC where requested. ISARIC also hosted a COVID-19 data platform where investigators and public health agencies from 82 countries shared and collaboratively analysed data from more than 950,000 individual patient records collected using the forms. These accumulated data were used to plan health resources, design interventional research studies, support medicine licensing applications, improve clinical care, and inform public health policy by identifying risk factors for severe illness [22,23,24,25,26,27,28]. The scale of this global effort represents a significant milestone in global pandemic collaboration, meriting in-depth analysis to learn from this experience and improve approaches for future outbreaks.

ISARIC and WHO continue to collaborate on the development of CRFs to characterise emerging infectious diseases. To date, the most commonly method used to design the ISARIC-WHO forms and similar CRFs relies on a review of the available evidence and an expert consensus process [20,29,30,31,32,33]. While this approach is valuable, the resulting forms are not commonly evaluated post hoc to determine and improve their performance. For the purpose of informing a more efficient design of forms for future outbreaks, we analysed the use and completion of the ISARIC-WHO COVID-19 CRF. The objective of the analysis was to assess the utilisation of each data field by identifying which fields were included or omitted across the locally adapted and/or implemented CRFs and to determine which fields were most reliably completed with patient data. Using COVID-19 patient data submitted to the ISARIC platform, the field utilisation in data from patients with severe disease was compared to the field utilisation in all patients. Understanding the patterns of field selection and entry provides a data-driven approach to refining the ISARIC-WHO form to optimise data collection strategies for other emerging health threats. The results of this analysis will inform how ISARIC, WHO, and outbreak research responders can structure future CRFs to streamline data capture for priority research questions.

## 2. Materials and Methods

### 2.1. Data Collection

The ISARIC-WHO COVID-19 CRF and ISARIC-WHO Clinical Characterisation Protocol were designed, adapted, and implemented for prospective data collection on hospitalised patients suspected or confirmed to have COVID-19. Recruitment strategies were defined at a site or national level, with suggested approaches to limiting recruitment bias offered by ISARIC (see Appendix A and Appendix B). The protocol, informed consent forms, and CRFs are available on the ISARIC website [34]. Decisions regarding which forms to use and how to adapt them were made at an institutional or national level.

Pseudonymised individual patient-level data collected between 24 January 2020 and 20 March 2023 were shared with the ISARIC COVID-19 Data Platform for the purpose of collaborative analysis and evidence generation. The list of contributing sites and individuals is available on the ISARIC website (www.isaric.org accessed on 1 July 2024). Detailed methods of data collection and curation have been described previously [22]. Data that were not mapped to the central database were not included in this analysis. Data collection sites implemented data quality checks according to locally available resources. ISARIC verified data submitted to the COVID-19 Data Platform for missing and expected values and queried discrepancies with the sites. Data have been analysed extensively in collaborative studies by the ISARIC partners, driving iterative review and the validation of all datasets by those who generated the data.

Severe patients were defined as those with any of the following: admission to an intensive or high-dependency care unit; and treatment with inotropes, vasopressors, high-flow nasal cannulas, invasive mechanical ventilation, or non-invasive mechanical ventilation. The total, median, and interquartile ranges were determined for the number of sites, total patients, severe patients, and number of collection months of each institutional dataset, and across all datasets.

### 2.2. Description of CRF Sections and Fields

**Figure 1:**
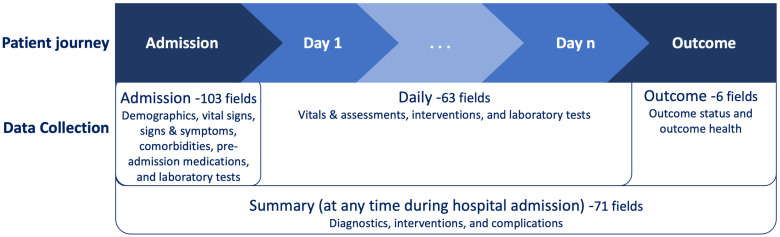
Data collection flow.

The ISARIC-WHO COVID-19 CRF has 243 fields divided across those collected at hospital admission (103 fields), ‘daily’ fields collected routinely each day during hospitalisation (63 fields), ‘summary’ fields collected on key events throughout hospitalisation (71 fields), and ‘outcome’ fields collected at death or discharge (6 fields) (Figure 1). The CRF fields are divided into groups based on the types of information they contain. Admission field groups include demographics, vital signs, signs and symptoms, comorbidities, pre-admission medications, and laboratory tests. Daily field groups include vitals and assessments, interventions, and laboratory tests. Diagnostics, interventions, and complications comprise the summary field groups. Outcome fields consist of outcome status (e.g., death, discharge, hospital transfer, and date) and outcome health (e.g., delayed discharge and ongoing care). The CRF, colour-coded with the results of this study, is available in the Supplementary Materials.

### 2.3. Evaluation of Field Inclusion and Completion

All fields on the ISARIC-WHO COVID-19 CRF (version 8DEC2021) were evaluated in this analysis, except for those added more than 12 months after the launch of the CRF (e.g., vaccination and virus variants), fields relevant to a particular subgroup of the population (e.g., infants), and fields that were not mapped to the central database (e.g., imaging). These fields were omitted from this analysis due to the absence of data in most patient records. No imputations were made for missing data.

The inclusion of each field from the original version into the versions used by individual institutions was at the discretion of each institution. Fields were considered included in an institutional dataset if the field featured within the CRF or if any data on that field were available in the dataset. The number and percentage of fields included in each dataset were calculated for each section of the CRF, as per Equation (1). The mean (see Equation (2)) and standard deviation of the number of fields included in each group of fields and across all datasets were determined. The results were visualised in a heatmap.

(1) $$field\;inclusion=(\;\frac{number\;of\;institutional\;datasets\;that\;included\;the\;field}{number\;of\;institutional\;datasets})\times 100$$

(2) $$mean\;field\;group\;inclusion=\frac{\left(field\;1\;inclusion+field\;2\;inclusion+\ldots field\;n\;inclusion\right)}{number\;of\;fields\;in\;the\;group}$$

For each field included by each site, the number of unique patients with data recorded in the field was divided by the total number of patients (Equation (3)). This was averaged across all datasets that included the field to establish each mean field completion across all data (Equation (4)). Where fields were dependent on other information (e.g., if pregnant: gestational age in weeks was recorded), the denominator of the completion proportion calculation was the total number of patients for whom the dependent information was true. Fields on the ‘daily’ section of the CRF were considered completed if they contained data for at least one day during admission. The mean field group completion was calculated across the datasets as per Equation (5).

(3) $$field\;completion\;dataset\;1=(\;\frac{number\;of\;dataset\;1\;patients\;with\;data\;in\;the\;field}{number\;of\;dataset\;1\;patients\;}\;)\times 100$$

(4) $$mean\;field\;completion=\frac{\left(field\;completion\;dataset\;1+field\;completion\;dataset\;2+\ldots field\;completion\;dataset\;n\right)}{number\;of\;datasets\;that\;include\;the\;field}$$

(5) $$mean\;field\;group\;completion=\frac{\left(mean\;field\;1\;completion+mean\;field\;2\;completion+\ldots mean\;field\;n\;completion\right)}{number\;of\;fields\;in\;the\;group}$$

### 2.4. Data Utilisation

Utilisation was quantified by calculating the Euclidean distance from an ideal point of (100, 100), representing the best case scenario of 100% completion proportion and 100% inclusion proportion. A greater distance from the ideal point indicates a reduced utilisation of a field, reflecting either less inclusion across sites, lower completeness, or both. Distances, hereafter called utilisation distances, for each field were calculated with all patient data using the mean proportion completed as the x coordinate and the mean proportion included as the y coordinate for each field (Equation (6)). Utilisation distances were normalised to a maximum of 100 by division with the highest distance, as per Equation (7), enabling comparison across fields.

(6) $$individual\;field\;utilisation\;distance=\sqrt{{\left(100-x\right)}^{2}+{\left(100-y\right)}^{2}}\;x=\mathrm{mean}\;\mathrm{field}\;\mathrm{completion},\;y=\mathrm{field}\;\mathrm{inclusion}$$

(7) $$normalised\;utilisation\;distance=\left(\;\frac{field\;utilisation\;distance}{\;\sqrt{100^{2}+100^{2}}}\right)\times 100$$

Calculations were repeated using data from severe patients only. The utilisation difference between all patients and the severe cohort was determined. Results were represented in a scatter plot.

Statistical analyses were performed using Python programming language (version 3.8.8), with the pandas library (version 1.2.4) [35,36].

## 3. Results

**Figure 2:**
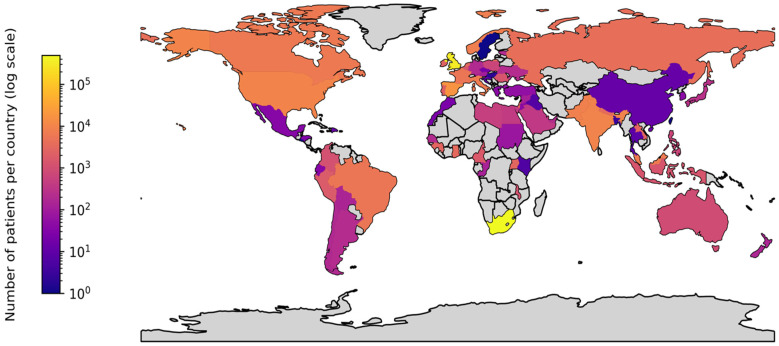
Map of data sources and volumes.

A total of 1886 hospitals belonging to 42 institutions, networks, or public health agencies contributed data to ISARIC’s COVID-19 data platform. Clinical characterisation data on 950,064 suspected or confirmed COVID-19 patients from 82 countries were included (Figure 2). Datasets varied in size and scope. This range spanned 19 single-site collection efforts, a multinational network of 258 sites across 44 countries, and a national clinical surveillance program across 627 sites. Data collection began before the peak case numbers in any participating country, with a median start date of 17 February 2020, and lasted for more than a year in most sites (median 16.8 months, IQR 7.6–28.1 months of data collection) (Table 1 and Table S1 in the Supplementary Materials). Across participating sites, 2155 staff were recorded as contributors to this data collection in the ISARIC database.

The proportion of severe patients included in each dataset ranged from 0% to 100% as datasets were collected in disparate settings, such as a district health centre in Maferenya, Guinea, and the intensive care unit of an academic medical centre in Karachi, Pakistan.

### 3.1. Field Group Inclusion

**Figure 3:**
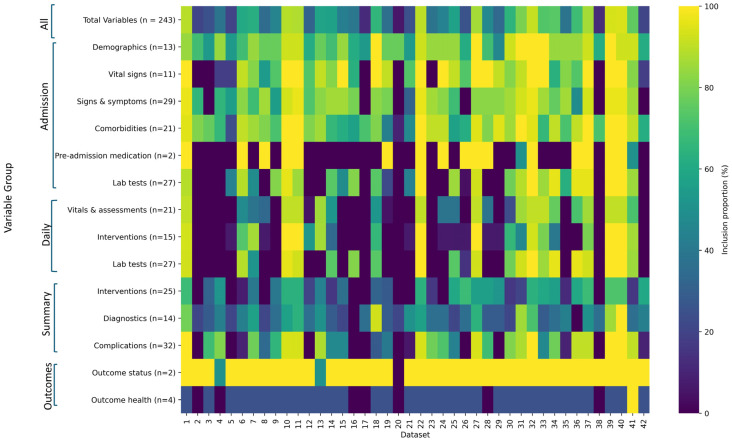
Heatmap of the ISARIC-WHO COVID-19 Case Report Form field group inclusion in institutional datasets. The inclusion proportion of each field group is visualised for 42 institutional datasets. Darker colours indicate a lower inclusion proportion.

Out of 243 fields, a mean of 129.6 fields (SD 64.4) were included in the CRFs implemented across the institutions (Table 2). Fields collected at outcome and hospital admission were most often included. Outcome status fields had the highest mean field group inclusion rates (1.9/2, 95.2%), followed by the admission group fields of demographics (10.3/13, 79.1%), comorbidities (16.4/21, 77.9%), vital signs (7.7/11, 70.3%), and signs and symptoms (20.0/29, 68.9%). Other fields were less often included on the institutional CRFs. On average, laboratory test fields were more often included on admission forms than daily forms (51.1% vs. 44.3%) and intervention fields were more often included on daily forms than summary forms (39.0% vs. 35.5%). A total of 13/42 datasets (31.0%) that collected summary intervention fields collected only 1 or 0 daily intervention fields. Outcome health fields had the lowest mean inclusion of 0.9/4 (22.6%) (Figure 3).

Some field groups were omitted completely from several datasets. Pre-admission medications fields did not appear in 24/42 (57.1%) of databases. A total of 11/42 (26.2%) datasets had no daily data collection fields. Data for field group inclusion of individual datasets are available in Table S2.

### 3.2. Field Group Completion

The ranking of completion proportion by data field group was similar to that of the inclusion proportion. Demographics and outcome status had the highest mean completion values of 91.6% and 89.8%, respectively. The remaining admission field groups were the next highest, ranging from 79.0% completion of comorbidities fields to 69.1% of vital signs fields. Outcome health fields had the lowest mean completion (19.9%), followed by laboratory test fields at admission (37.0%) and daily (39.3%) (Table 2).

There was no appreciable difference in field inclusion between all patients and severe patients (mean decrease of 0.9% inclusion), nor in the completion of the most highly included field groups of demographic and outcome status data. However, the mean completion was higher in severe patients for all other field groups. Daily data were more often entered for severe patients, resulting in the largest increase in completion; intervention, laboratory tests, and vitals and assessment mean field group completion increased by 23.4%, 13.3%, and 11.8%, respectively. Complication fields had a mean increase of 12.6% more completion in severe patients (Table 2).

### 3.3. Field Group Utilisation

The utilisation distance, assigned to express proximity to a point of 100% inclusion and completion, followed the field group trends of its components, with lower scores demonstrating a higher utilisation. The mean utilisation distance across all 243 data fields was 43.9 (SD 21.0) in all patients and 41.0 (SD 19.9) in severe patients. Field groups with the lowest mean utilisation distance in all patients were outcome status (8.0, SD 3.3), demographics (16.3, SD 0.14), comorbidities (22.9, SD 13.8), signs and symptoms (27.2, SD 14.4), and vital signs (30.6, SD 10.8). Summary comorbidities had a mean utilisation distance of 41.8 (SD 12.5). Field groups collected daily were all higher than the mean utilisation distance, as were admission laboratory tests, summary diagnostics and interventions, and outcome health fields in all patients (Table 2). This ranking of field group utilisation distance was consistent in severe patients.

### 3.4. Individual Field Utilisation

**Figure 4:**
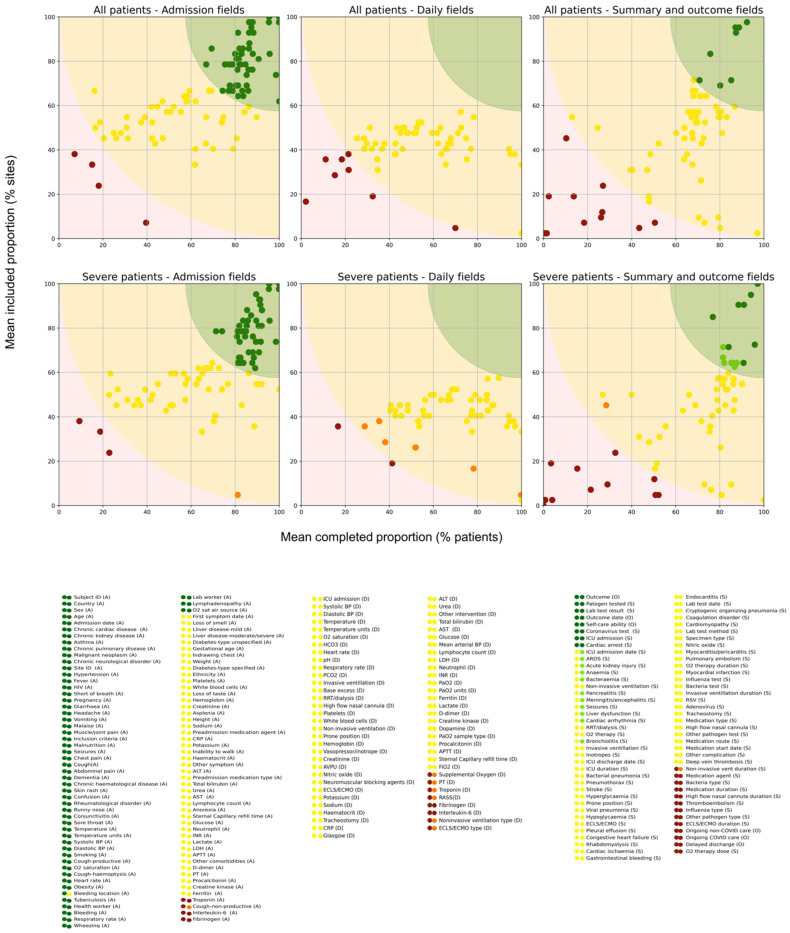
Utilisation distance of data fields in the ISARIC-WHO COVID-19 Case Report From for all (N = 950,064) and severe (N = 256,529) patient data. Utilisation distance comprises the mean completion rate (x-axis) and inclusion rate (y-axis) among institutions. Colours are assigned to dots and backgrounds as visual cues, marking utilisation distances lower than 30 as dark green, those from 30 to 70 as yellow, and those greater than 70 as red. Colours in the severe patient plots are varied to indicate a decrease across this threshold from all patients to severe patients: light green for fields with utilisation distances that decreased to below 30 in severe patients and orange for fields with utilisation distances that decreased to below 70 in severe patients. Field labels are listed from the lowest to the highest utilisation distance within each collection time: (A) admission (**left**), (D) daily (**centre**), and (S) summary and (O) outcome (**right**). Next to each field name, the left circle applies to ‘All patients’, and the right circle applies to ‘Severe patients’.

The utilisation distances of individual fields highlight the variability of results within a group and identify specific fields that impact group utilisation. Subject IDs were assigned to all patients and were therefore included and fully completed in all CRFs (utilisation distance 0). Country was defined in the metadata of each dataset and was therefore included and complete for 100% of patients (utilisation distance 0). The next lowest utilisation distance in both cohorts included variables for sex (1.7), age (3.2), admission date (3.7), and outcome (5.7 in all patients and 2.0 in severe patients). Pathogen test results (8.7 in all and 9.3 in severe for pathogen type, and 9.7 in all and 10.6 in severe for test result) were additionally in the lowest utilisation distances. Other fields in the low (green) range of utilisation distances included comorbidities, signs and symptoms at admission, vital signs at admission, and additional outcome information. The central (yellow) range of utilisation distances were dominated by complications, interventions, vitals and assessments, and laboratory tests. The highest (red) of utilisation distances included fields for less common laboratory or diagnostic tests, outcome health, and details (type or duration) of interventions. Fields that capture features of severe illness feature more in the higher range of utilisation distances within each group (Figure 4).

The utilisation distance was lower in the severe patient cohort than in all patients, with a mean decrease in utilisation distance of 3.0 (SD 1.2) across all fields. Decreases were driven by higher completion proportions in the severe population. Daily interventions, daily laboratory tests, summary complications, and outcome status field groups had the largest decreases in utilisation distance (7.8, 6.3, 4.3, and 4.3, respectively). Individual fields associated with critical care were more likely to be included and/or completed in the severe patient cohort than in all patients. The largest decreases in utilisation distance were in fields for treatments targeting severe disease: extracorporeal membrane oxygenation type (30.0), dopamine treatment (19.4), non-invasive ventilation type (20.3) and duration (10.6), and high-flow nasal cannula duration (9.5). Examples of individual utilisation distances are presented in Table 3, with the utilisation colouring matching that in Figure 4. Utilisation scores for all fields are available in Table S3, in the Supplementary Materials.

## 4. Discussion

The implementation of the ISARIC-WHO COVID-19 CRF, or its derivatives, has been evaluated in 42 distinct data collection initiatives across 1886 hospitals. Despite their differences in geography, context, data collection periods, and volume of data collection, consistent patterns of data field inclusion and completion are observed across these institutions.

### 4.1. Field Inclusion

Of the 243 fields included on the ISARIC-WHO COVID-19 form, the mean number of fields included in CRFs adapted and implemented by ISARIC partners is 129.6 (53.3%). The low mean field inclusion resulted in high proportions of missing data across the aggregate data, particularly in the outcome health, laboratory, and intervention field groups. This restricted the populations that could be included in cross-institutional analyses, limiting the power and generalisability of the results to inform clinical and public health decision making.

Data collection, and the adaptation and/or implementation of the CRF used, began early in the outbreak. The majority of sites had already initiated data collection before the WHO declared COVID-19 a pandemic on 11 March 2020 [37]. Therefore, decisions about which variables to include in the institutional forms were made before the magnitude of COVID-19’s impact was known. Motivations to reduce the number of fields included in the institutional forms may have included limitations of infrastructure, patient management, data flow, human resources, and staff motivation.

The low inclusion of intervention fields (39.0% and 35.5% on the daily and summary forms, respectively) may be due to a lack of equipment for higher-level care, such as different types of ventilation, dialysis, or extracorporeal membrane oxygenation [38]. Several participating sites in low-resource settings did not have access to these interventions. The omission of laboratory fields to align with patient management guidelines is a practical decision when tests, such as that for interleukin-6 (38.1% and 35.7% included in the admission and daily forms, respectively), are not part of standard care [39].

Data flow patterns limited the inclusion of fields in institutional data collection when the source of data used to complete the CRF did not cover all CRF fields. For example, when electronic health record data extraction is used to populate the CRF, the information required for some data fields may not be available in the records [40]. In other cases, it may not be possible to adapt pre-existing data systems such as patient registries to accommodate all data in the CRF. Some sites reported reducing fields that required clinical judgement as data collection was undertaken by data staff without clinical training. Other sites omitted fields that required data that were not available in medical records [41]. These factors are likely contributors to the low inclusion of fields on outcome health (3/4 fields had only 2.4% inclusion).

Completing and entering data for the entire ISARIC-WHO COVID-19 CRF was reported to take approximately 30 min per patient when inpatient stays are limited, and a local ethics committee waived the requirement for written informed consent [42]. Bandwidth limitations may extend this time for electronic data capture. During a developing public health emergency, human resource constraints due to caseloads and/or staff illness may lead to conservative research data collection approaches despite the need for evidence. Two nation-wide research programs that contributed to the ISARIC COVID-19 Data Platform illustrate this. In the United Kingdom, the ISARIC4C study included 75.7% of the CRF fields to collect data on >300,000 patients in 323 hospitals under the direction of national health authorities who mobilised dedicated research staff to collect data in all hospitals [17,43]. In South Africa, the National Institute of Communicable Diseases conducted a nationwide hospital surveillance and research study across 627 hospitals. Without dedicated research staff available, the decision was made to reduce the ISARIC-WHO CRF down to a feasible 20.6% of the original fields, which were collected for >490,000 patients [19,44]. These findings highlight the value of dedicated research staff who can be seconded for outbreak research response when needed.

Though differing operational limitations define institutional field inclusions and exclusions, the overall inclusion trend indicates alignment in research priorities across the institutions. Outcome status fields and key admission fields (demographics, comorbidities, vital signs, and signs and symptoms) had the highest inclusion rates across the institutions (68.9–95.2%). The focus on these time points is supported by scientific justification from several sources, including Harris et al.’s 2020 review of key early outbreak research questions for SARS-CoV and MERS-CoV [45], and Rojek et al.’s list of minimal observational data needed to design clinical trials for high-priority pathogens [1]. These highlight clinical presentation, risk factors for death and severe illness, optimal diagnostics, case defining features, risk factors for infection, and outcomes as research priorities. All of these can be addressed by information collected at admission and/or outcome. The WHO’s 2020 Strategic Preparedness and Response Plan for the Novel Coronavirus echoes this inventory of priorities with a research focus on disease severity and identifying at-risk groups [46].

### 4.2. Field Completion

Unlike the institutional decisions that drove inclusion decisions, the completion of data fields relied on individual clinical and research staff. The completion of fields is subject to operational barriers when institutional decisions cannot account for all circumstances. In addition, independent decisions can be made by the individuals responsible for data collection. Though pre-admission medication fields were included on 61.9% of forms, they were completed just 40.5% of the time. This indicates a likely absence of information on the data source, and/or limitations of staff time to complete all fields.

Fields related to outcome health had consistently low completion rates across institutions (19.9% mean completion for all patients). This information regarding the health needs of the patient post-discharge may not have been assessed or available at the point of outcome, or it may have been perceived as low value or outside of the responsibilities of those collecting these data. Though many studies were later executed to understand long COVID, the absence of these data in ISARIC’s pioneering characterisation work prevented the opportunity for earlier detection of sequela. Laboratory tests had the next lowest completion rates (37.0% and 39.3% for admission and daily data in all patients, respectively), likely due to many parameters not being measured as a part of clinical care. This is supported by the increase in completion for severe patients (44.9% and 52.6% at admission and daily, respectively), whose health complications would warrant additional laboratory monitoring.

### 4.3. Adjusting for Severity

Increased data completion for severe patients accounted for a higher field utilisation in most field groups. Additional data may enable an understanding of the mechanisms of severe disease and inform effective treatment. However, without a systematic severity-based collection strategy, individual staff may apply ad hoc approaches based on perceived relevance of data in higher-level care and/or increased availability of information due to more frequent monitoring. Prospective planning of research questions and the data required to address them is needed to promote efficiency in evidence generation.

The additional data required to understand severe disease should be collected only in the relevant patients. The consistency of field inclusion between all patients and severe patients reflects institutional-level decisions to use the same CRF across all levels of severity. The practicality of this decision highlights the importance of CRFs that can adapt to different data requirements where relevant to the severity of illness.

### 4.4. Limitations

The data used in this analysis were collected from diverse institutions with varying levels of resources and capacities. This diversity, while providing a broad perspective, does not represent all settings in which research on emerging infectious diseases is important. The ISARIC collaboration relied on voluntary participation and data sharing, which may introduce selection bias for institutions with interest, resources, and an enabling regulatory framework for these activities. This bias may predispose the inclusion and completion results of participating institutions to be higher than a true average of all contexts. When planning data collection that requires robust representation, it is important to make provisions to include health care facilities that lack research resources. The interpretation of clinical evidence generated only from sites with research resources should also consider potential biases in patient outcomes.

Differences over time or between different settings were not explored in this analysis. Both may have been drivers of change in completion rates. Additionally, as the data are all from patients with COVID-19, differences in research priorities for different emerging syndromes may require the collection of data on different fields and/or events. These should be considered based on the context and state of knowledge and can consider how the results of this study may be applied.

While we identified fields and field groups with lower utilisation based on inclusion and completion rates, these metrics do not fully capture the clinical significance or research value of individual data fields. Some fields may be underreported yet hold critical importance for specific populations, research questions, clinical decisions, or health policy.

### 4.5. Towards Optimising Clinical Epidemiology in Disease Outbreaks

Ensuring that research resources are used efficiently is important to maximise the improvement in patient outcomes in all diseases. In the context of emerging infectious diseases with limited evidence on effective interventions, this efficiency is particularly important to inform a rapid and reliable response that reduces the impact of the outbreak. Additionally, by minimising the time required for data collection, health care workers can dedicate more attention to providing front-line patient care.

The results of this study inform several key efficiency-focused improvements to CRFs for the clinical characterisation of diseases in future outbreaks. Guided by the utilisation of each field, planned changes for future ISARIC-WHO CRFs include the modularisation of contents, operational streamlining in field selection, and iterative review based on accruing evidence. These approaches will enhance the clinical and public health values of the data collected and improve data capture efficiency.

CRFs should focus on locally prioritised research and public health questions, designed with incremental modules that can be completed or omitted as resources allow. Sites with limited data collection resources can complete essential fields capturing key case-defining features, the spectrum of clinical presentation, risk factors, laboratory diagnosis, and outcomes. Sites with more resources can include additional fields for a more comprehensive understanding of the disease spectrum. A third module of daily data collection can be available for capable sites, generating detailed evidence on the disease course, patient journey, and specific populations. Fields relevant only to research questions on severe disease or patients in critical condition should be in a standalone module, used only for patients who achieve a threshold of severity.

This tiered approach ensures all sites focus their data collection capacity on the knowledge gaps most relevant to their patients. Sites may adjust the number of modules based on caseload, resource availability, or other factors as they change during an outbreak. It is important to understand and account for potential bias in any approach that focuses on well-resourced centres.

Fields that cannot be completed at a hospital should be excluded and documented to support the understanding of missing values. This allows researchers to make informed decisions about appropriate methods for handling the missing information, address potential biases, and interpret the results.

To ensure the relevance and comprehensiveness of the CRF, dynamic updates should be made based on the continuous review of research priorities and data utilisation monitoring employing the methods described in this paper. By maintaining flexibility and responsiveness in data collection processes, CRFs can support efficient clinical characterisation and enhance the quality and usability of collected data during outbreaks.

## 5. Conclusions

A data-driven approach to designing CRFs for the clinical characterisation of emerging infectious diseases can enhance data collection efficiency to inform patient care and public health response. The selection of data collection fields should always be tailored to prioritised research objectives, the outbreak dynamics, and the context of local health systems. The availability of tiered, standardised, template CRFs that optimise the inclusion of relevant data fields can accelerate the generation of critical evidence. The adoption of standard data variables and modular options additionally promotes the alignment of global data collection efforts, offering further opportunity to reduce time to evidence through aggregate analysis that impacts policy. These principles can increase the efficiency of evidence generation in many types of exploratory data collection. ISARIC and WHO will continue to develop CRFs for emerging infectious diseases, based on locally and globally defined research priorities and the practical insights gained from their actual use.

## Figures and Tables

**Table 1:** Site and patient distribution across 42 institutional datasets included in the ISARIC COVID-19 Data Platform.

	Hospitals	Countries	All Patients	Severe Patients *	Data Collection Start Date	Months of Data Collection
Total	1886	82	950,064	256,529		725.7
Range	1–627	1–44	30–494,547	0–122,754	24/01/2020–17/03/2021	1.0–38.0
Median	1	1	1014.5	107	17/02/2020	16.8
IQR	1.0–28.0	1.0–1.0	182.8–4485.5	22.5–1147.8		7.6–28.1

* Severe patients are a subset of ‘All patients’.

**Table 2:** Utilisation distance of data field groups in all and severe patient cohorts.

Collection Time	Field Group	All Patient Data			Severe Patient Data			Difference
		Utilisation Distance Mean (SD)	Completion % Mean (SD)	Inclusion % Mean (SD)	Utilisation Distance Mean (SD)	Completion % Mean (SD)	Inclusion % Mean (SD)	Utilisation Distance Delta (SD)
Outcome	Outcome status	8.0 (3.3)	89.8 (3.5)	95.2 (3.4)	3.7 (2.4)	95.7 (2.1)	97.5 (3.5)	4.3 (0.8)
Admission	Demographics	16.3 (14.0)	91.6 (8.2)	79.1 (18.7)	16.3 (14.0)	91.6 (8.2)	79.1 (18.7)	0.0 (0.0)
Admission	Comorbidities	22.9 (13.8)	79.0 (16.1)	77.9 (15.8)	22.9 (13.7)	83.2 (15.8)	74.1 (15.1)	0.0 (0.1)
Admission	Signs and symptoms	27.2 (14.4)	78.4 (10.7)	68.9 (18.6)	27.1 (13.3)	82.3 (7.5)	66.7 (18.6)	0.0 (1.1)
Admission	Vital signs	30.6 (10.8)	69.1 (13.5)	70.3 (9.3)	28.5 (11.4)	75.0 (14.5)	69.0 (10.0)	2.0 (−0.7)
Summary	Complications	41.8 (12.5)	65.5 (12.9)	52.8 (14.9)	37.5 (13.4)	78.1 (14.6)	52.4 (14.9)	4.3 (−0.9)
Admission	Pre-admission medication	47.3 (3.8)	70.5 (12.2)	40.5 (0.0)	46.5 (0.6)	79.8 (12.7)	38.1 (3.4)	0.8 (3.3)
Daily	Vitals and assessments	51.6 (12.3)	64.5 (23.0)	39.6 (13.9)	47.5 (11.3)	76.3 (18.3)	39.2 (14.2)	4.0 (1.0)
Summary	Diagnostics	52.8 (25.0)	54.0 (24.8)	42.3 (28.2)	52.0 (24.1)	59.1 (24.9)	40.1 (26.4)	0.8 (0.9)
Daily	Interventions	53.1 (14.3)	59.1 (24.5)	39.0 (9.2)	45.3 (8.0)	82.5 (11.4)	39.1 (9.7)	7.8 (6.4)
Summary	Interventions	55.0 (21.5)	61.2 (28.2)	35.5 (23.1)	52.5 (21.6)	67.6 (26.9)	35.8 (23.9)	2.5 (−0.2)
Admission	Lab tests	56.5 (12.0)	37.0 (15.1)	51.1 (9.8)	52.7 (12.6)	44.9 (16.9)	50.4 (9.4)	3.8 (−0.6)
Daily	Lab tests	58.4 (9.1)	39.3 (12.2)	44.3 (7.4)	52.1 (9.7)	52.6 (13.9)	44.1 (7.3)	6.3 (−0.6)
Outcome	Outcome health	78.8 (38.6)	19.9 (37.2)	22.6 (40.5)	78.3 (39.2)	20.5 (37.6)	23.1 (41.2)	0.5 (−0.6)
All	All	43.9 (21.0)	61.6 (24.3)	53.3 (22.4)	41.0 (19.9)	70.2 (22.7)	52.4 (22.0)	3.0 (1.2)

The mean proportion of datasets that included the field and completed the field is expressed in %. The mean utilisation distance expresses the mean distance from 100% inclusion and 100% completion of all fields in a given group. Delta is the mean field group utilisation distance in all patients minus the mean field group utilisation distance in severe patients.

**Table 3:** Examples of utilisation distance of individual fields in all and severe patient cohorts.

Collection Time	Field Group	Field	Utilisation Distance, All Patients (x, y)	Utilisation Distance, Severe Patients (x, y)	Delta	Utilisation Colour Group (Severe)
Admission	Demographics	Sex	1.7 (99.9, 97.6)	1.7 (99.9, 97.6)	0	
Daily	Vitals and assessments	O_2_ saturation	42.3 (71.2, 47.6)	38.7 (84.1, 47.6)	3.6	
Outcome	Outcome status	Outcome	5.7 (92.3, 97.6)	2.0 (97.2, 100)	3.7	
Admission	Lab tests	INR	58.5 (30.8, 54.8)	54.6 (39.2, 52.4)	3.8	
Summary	Complications	Bacteraemia	32.4 (71.4, 64.3)	27.3 (85.3, 64.3)	5.0	
Daily	Lab tests	Troponin	73.4 (18.4, 35.7)	67.8 (28.8, 35.7)	5.6	
Summary	Complications	ARDS	30.3 (68.0, 71.4)	24.0 (81.6, 71.4)	6.3	
Daily	Lab tests	ALT	52.1 (45.9, 50.0)	45.8 (58.8, 50.0)	6.3	
Daily	Vitals and assessments	Glasgow coma score	51.9 (48.5, 47.6)	45.2 (63.5, 47.6)	6.8	
Daily	Lab tests	PT	70.7 (21.4, 38.1)	63.3 (35.4, 38.1)	7.5	
Daily	Lab tests	Lactate	63.7 (28.5, 45.2)	55.0 (44.8, 45.2)	8.7	
Daily	Vitals and assessments	PaO_2_ sample type	64.9 (34.4, 35.7)	56.0 (53.6, 35.7)	8.9	
Summary	Interventions	High-flow nasal cannula duration	81.1 (26.6, 11.9)	71.5 (50.3, 11.9)	9.5	
Summary	Interventions	Non-invasive vent duration	74.3 (10.3, 45.2)	63.7 (28.5, 45.2)	10.6	
Daily	Interventions	Dopamine	64.9 (34.5, 35.7)	45.5 (97.1, 35.7)	19.4	
Daily	Interventions	ECLS/ECMO type	90.9 (2.1, 16.7)	60.9 (78.2, 16.7)	30.0	

Fields listed are a random sample. Delta is the utilisation distance in all patients minus the utilisation distance in severe patients. Utilisation distance is a function of the mean field completion (x) and field inclusion (y). Utilisation colour groups in severe patients: light green indicates fields with utilisation distances that decreased to below 30 in severe patients; orange indicates fields with utilisation distances that decreased to below 70 in severe patients; dark green indicates fields with utilisation distances lower than 30 in all patients and severe patients; yellow indicates those from 30 to 70; and red indicates those greater than 70.

## Data Availability

The data that underpin this analysis are highly detailed clinical data on individuals hospitalised with COVID-19. Due to the sensitive nature of these data and the associated privacy concerns, they are available via a governed data access mechanism following review of a data access committee. Data can be requested via the IDDO COVID-19 Data Sharing Platform (http://www.iddo.org/covid-19 accessed on 1 July 2024). The Data Access Application, Terms of Access, and details of the Data Access Committee are available on the website. Briefly, the requirements for access are a request from a qualified researcher working with a legal entity who have a health and/or research remit and having a scientifically valid reason for data access that adheres to appropriate ethical principles. A small subset of sites who contributed data to this analysis have not agreed to pooled data sharing as above. In the case of requiring access to these data, please contact the corresponding author in the first instance who will look into facilitating access. The analysis code is available in the Supplementary Materials.

## Supplementary Materials

The following supporting information can be downloaded at https://www.mdpi.com/article/10.3390/epidemiologia5030039/s1: ISARIC-WHO COVID-19 CRF 8DEC21; Table S1. Site and patient distribution across 42 institutional datasets, Table S2. Field group inclusion of individual datasets, and Table S3. Utilisation distance of individual fields: analysis code.

## Appendix A. ISARIC-WHO Clinical Characterisation Protocol: Sampling Strategies to Reduce Bias

Robust analysis requires methodical selection of patients for data collection. Each site has its own requirements, abilities, and limitations to participant recruitment and should select the sampling approach that fits the best. Record the sampling approach taken for each unique date range in your site-level CRF.

**Type of Sampling**	**Explanation**
Census	Enrol all potential participants who present to your setting.
Sequential/systematic sampling	Enrol patients based on their time of presentation, e.g., every 3rd patient who presents to the hospital, or all patients who present on even calendar days (2nd, 4th, 6th).
Simple random sampling	Use a tool to randomly determine if each patient is enrolled, e.g., use an online tool to generate a random list of Yes/No variables in the desired proportion and apply them sequentially as patients present (note: this should be conducted so that no one knows the next variable).
Defined population census, e.g., ICU patients	Enrol all patients admitted to the ICU.

## Appendix B. ISARIC Clinical Characterisation Group

Sabriya Abdalasalam, Alaa Abdalfattah Abdalhadi, Walaa Abdalla, Naana Reyam Abdalla, Almthani Hamza Abdalrheem, Ashraf Abdalsalam, Saedah Abdeewi, Esraa Hassan Abdelgaum, Mohamed Abdelhalim, Mohammed Abdelkabir, Sheryl Ann Abdukahil, Lamees Adil Abdulbaqi, Widyan Abdulhamid, Salaheddin Abdulhamid, Nurul Najmee Abdulkadir, Eman Abdulwahed, Rawad Abdunabi, Ryuzo Abe, Laurent Abel, Ahmed Mohammed Abodina, Khaled Abouelmagd, Amal Abrous, Kamal Abu Jabal, Nashat Abu Salah, Salsabeel M. A. Abukhalaf, Abdurraouf Abusalama, Tareg Abdallah Abuzaid, Subhash Acharya, Andrew Acker, Safia Adem, Manuella Ademnou, Francisca Adewhajah, Neill KJ Adhikari, Diana Adrião, Samuel Yaw Adu, Anthony Afum-Adjei Awuah, Melvin Agbogbatey, Saleh Al Ageel, Musaab Mohammed Ahmed, Aya Mustafa Ahmed, Shakeel Ahmed, Zainab Ahmed Alaraji, Abdulrahman Ahmed Elhefnawy Enan, Reham Abdelhamid Ahmed Khalil, Ali Mostafa Ahmed Mohamed Abdelaziz, Marina Aiello, Kate Ainscough, Eka Airlangga, Tharwat Aisa, Ali Aisha, Bugila Aisha, Ali Ait Hssain, Takako Akimoto, Ernita Akmal, Chika Akwani, Eman Al Qasim, Yasmina Alaa, Ahmed Alajeeli, Ahmed Alali, Razi Alalqam, Aliya Mohammed Alameen, Mohammed Al-Aquily, Zinah A. Alaraji, Khalid Albakry, Safa Albatni, Angela Alberti, Tala Al-dabbous, Amer Aldhalia, Abdulkarim Aldoukali, Marta Alessi, Beatrice Alex, Kévin Alexandre, Abdulrahman Al-Fares, Asil Alflite, Huda Alfoudri, Khadeejeh M. A. Alfroukh, Qamrah Alhadad, Hoda Salem Alhaddad, Maali Khalid Mohamed Abdalla Alhasan, Ahmad Nabil Alhouri, Hasan Alhouri, Adam Ali, Maha TagElser Mohammed Ali, Imran Ali, Syed Ali Abbas, Yomna Ali Abdelghafar, Naseem Ali Shah, Kazali Enagnon Alidjnou, Mahmoud Aljadi, Sarah Aljamal, Mohammed Alkahlout, Khalid Jehad Khalid Alkaraki, Akram Alkaseek, Qabas Alkhafajee, Clotilde Allavena, Nathalie Allou, Lana Almasri, Abdulrahman Almjersah, Raja Ahmed Alqandouz, Walaa Alrfaea, Moayad Alrifaee, Rawan Alsaadi, Yousef Al-Saba’a, Entisar Alshareea, Eslam Alshenawy, Aneela Altaf, Rita Alves, João Melo Alves, João Alves, Joana Alves Cabrita, Maria Amaral, Amro Essam Amer, Nur Amira, Heidi Ammerlaan, Amos Amoako Adusei, John Amuasi, Roberto Andini, Margarita Andreeva, Claire Andrejak, Andrea Angheben, François Angoulvant, Sophia Ankrah, Séverine Ansart, Massimo Antonelli, Carlos Alexandre Antunes de Brito, Kazi Rubayet Anwar, Ardiyan Apriyana, Yaseen Arabi, Irene Aragao, Francisco Arancibia, Carolline Araujo, Antonio Arcadipane, Patrick Archambault, Lukas Arenz, Jean-Benoît Arlet, Christel Arnold-Day, Ana Aroca, Rakesh Arora, Lovkesh Arora, Elise Artaud-Macari, Diptesh Aryal, Motohiro Asaki, Angel Asensio, Elizabeth A. Ashley, Muhammad Ashraf, Muhammad Sheharyar Ashraf, Abir Ben Ashur, Franklin Asiedu-Bekoe, Namra Asif, Mohammad Asim, Grace Assi, Jean Baptiste Assie, Amirul Asyraf, Ahmed Atia, Minahel Atif, Asia Atif Abdelrhman Abdallahrs, Anika Atique, Moad Atlowly, AM Udara Lakshan Attanyake, Johann Auchabie, Hugues Aumaitre, Adrien Auvet, Sergey Avdeev, Abdelmalek Awad Ali Mohammed, Eyvind W. Axelsen, Ared Ayad, Ahmed Ayman Hassan Helmi, Laurène Azemar, Mohammed Azizeldin, Cecile Azoulay, Hakeem Babatunde, Benjamin Bach, Delphine Bachelet, Claudine Badr, Roar Bævre-Jensen, Nadia Baig, John Kenneth Baillie, J Kevin Baird, Zinah Aqeel Abdulzahra Bairmani, Erica Bak, Agamemnon Bakakos, Nazreen Abu Bakar, Hibah Bileid Bakeer, Ashraf Bakri, Andriy Bal, Mohanaprasanth Balakrishnan, Irene Bandoh, Firouzé Bani-Sadr, Renata Barbalho, Nicholas Yuri Barbosa, Wendy S. Barclay, Saef Umar Barnett, Michaela Barnikel, Helena Barrasa, Cleide Barrigoto, Marie Bartoli, Cheryl Bartone, Joaquín Baruch, Romain Basmaci, Muhammad Fadhli Hassin Basri, AbdAlkarim Batool, Denise Battaglini, Jules Bauer, Diego Fernando Bautista Rincon, Denisse Bazan Dow, Abigail Beane, John Beca, Alexandra Bedossa, Ker Hong Bee, Husna Begum, Albertus Beishuizen, Aleksandr Beljantsev, David Bellemare, Anna Beltrame, Beatriz Amorim Beltrão, Marine Beluze, Nicolas Benech, Lionel Eric Benjiman, Suzanne Bennett, Luís Bento, Jan-Erik Berdal, Lamis Berdeweel, Delphine Bergeaud, Hazel Bergin, José Luis Bernal Sobrino, Kikaire Bernard, Giulia Bertoli, Lorenzo Bertolino, Simon Bessis, Adam Betz, Sybille Bevilcaqua, Karine Bezulier, Amar Bhatt, Krishna Bhavsar, Isabella Bianchi, Claudia Bianco, Sandra Bichoka, Farah Nadiah Bidin, Felwa Bin Humaid, Mohd Nazlin Bin Kamarudin, Muhannud Binnawara, Zeno Bisoffi, Patrick Biston, Laurent Bitker, Mustapha Bittaye, Jonathan Bitton, Pablo Blanco-Schweizer, Aaron Blandino Ortiz, Catherine Blier, Frank Bloos, Mathieu Blot, Lucille Blumberg, Polina Bobkova, Filomena Boccia, Laetitia Bodenes, Debby Bogaert, Anne-Hélène Boivin, Ariel Bolanga, Isabela Bolaños, Pierre-Adrien Bolze, Patrizia Bonelli, Aurelius Bonifasius, Joseph Bonney, Diogo Borges, Raphaël Borie, Hans Martin Bosse, Elisabeth Botelho-Nevers, Lila Bouadma, Olivier Bouchaud, Sabelline Bouchez, Kévin Bouiller, Laurence Bouillet, Camile Bouisse, Latsaniphone Bountthasavong, Anne-Sophie Boureau, John Bourke, Maude Bouscambert, Aurore Bousquet, Marielle Boyer-Besseyre, Maria Boylan, Fernando Augusto Bozza, Axelle Braconnier, Timo Brandenburger, Filipa Brás Monteiro, Luca Brazzi, Patrick Breen, Dorothy Breen, David Brewster, Kathy Brickell, Christopher Brighting, Tessa Broadley, Helen Brotherton, Alex Browne, Nicolas Brozzi, Sonja Hjellegjerde Brunvoll, Marjolein Brusse-Keizer, Petra Bryda, Nina Buchtele, Polina Bugaeva, Marielle Buisson, Danilo Buonsenso, Erlina Burhan, Donald Buri, Aidan Burrell, Ingrid G. Bustos, Denis Butnaru, André Cabie, Susana Cabral, Joana Cabrita, Eder Caceres, Cyril Cadoz, Rui Caetano Garcês, Mia Callahan, Kate Calligy, Jose Andres Calvache, Caterina Caminiti, João Camões, Paul Campbell, Josie Campisi, Cecilia Canepa, Mireia Cantero, Janice Caoili, Pauline Caraux-Paz, Sheila Cárcel, Sofia Cardoso, Nelson Cardoso, Filipa Cardoso, Filipe Cardoso, Simone Carelli, Francesca Carlacci, Nicolas Carlier, Thierry Carmoi, Gayle Carney, Inês Carqueja, Marie-Christine Carret, François Martin Carrier, Ida Carroll, Leonor Carvalho, Maire-Laure Casanova, Mariana Cascão, Siobhan Casey, José Casimiro, Bailey Cassandra, Silvia Castañeda, Nidyanara Castanheira, Guylaine Castor-Alexandre, Ivo Castro, Ana Catarino, François-Xavier Catherine, Roberta Cavalin, Giulio Giovanni Cavalli, Alexandros Cavayas, Adrian Ceccato, Masaneh Ceesay, Shelby Cerkovnik, Minerva Cervantes-Gonzalez, Muge Cevik, Anissa Chair, Catherine Chakveatze, Adrienne Chan, Meera Chand, Jean-Marc Chapplain, Charlotte Charpentier, Julie Chas, Muhammad Mobin Chaudry, Jonathan Samuel Chávez Iñiguez, Anjellica Chen, Yih-Sharng Chen, Léo Chenard, Matthew Pellan Cheng, Antoine Cheret, Alfredo Antonio Chetta, Thibault Chiarabini, Julian Chica, Suresh Kumar Chidambaram, Leong Chin Tho, Catherine Chirouze, Davide Chiumello, Hwa Jin Cho, Sung-Min Cho, Bernard Cholley, Danoy Chommanam, Marie-Charlotte Chopin, Ting Soo Chow, Nathaniel Christy, Hiu Jian Chua, Jonathan Chua, Jose Pedro Cidade, José Miguel Cisneros Herreros, Barbara Wanjiru Citarella, Anna Ciullo, Jennifer Clarke, Rolando Claure-Del Granado, Sara Clohisey, Cassidy Codan, Caitriona Cody, Jennifer Coles, Gwenhaël Colin, Michael Collins, Jennifer Connolly, Marie Connor, Anne Conrad, Elaine Conway, Graham S. Cooke, Hugues Cordel, Amanda Corley, Sabine Cornelis, Alexander Daniel Cornet, Arianne Joy Corpuz, Andrea Cortegiani, Grégory Corvaisier, Sandrine Couffin-Cadiergues, Roxane Courtois, Stéphanie Cousse, Juthaporn Cowan, Rachel Cregan, Charles Crepy D’Orleans, Cosimo Cristella, Gloria Crowl, Jonathan Crump, Claudina Cruz, Juan Luis Cruz Bermúdez, Jaime Cruz Rojo, Marc Csete, Alberto Cucino, Ailbhe Cullen, Matthew Cummings, Gerard Curley, Elodie Curlier, Colleen Curran, Paula Custodio, Ana da Silva Filipe, Charlene Da Silveira, Al-Awwab Dabaliz, Andrew Dagens, John Arne Dahl, Darren Dahly, Umberto D’Alessandro, Peter Daley, Zaina Dalloul, Heidi Dalton, Jo Dalton, Seamus Daly, Juliana Damas, Joycelyn Dame, Federico D’Amico, Cammandji Damien, Hegazy Dana, Nick Daneman, Jorge Dantas, Frédérick D’Aragon, Menno de Jong, Gillian de Loughry, Diego de Mendoza, Etienne De Montmollin, Ra De Pablo, Ana Isabel de Pinho Oliveira, Rosanna De Rosa, Cristina De Rose, Thushan de Silva, Jillian Deacon, David Dean, Alexa Debard, Bianca DeBenedictis, Marie-Pierre Debray, Nathalie DeCastro, William Dechert, Romain Decours, Eve Defous, Isabelle Delacroix, Alexandre Delamou, Karen Delavigne, Nathalie M. Delfos, Andrea Dell’Amore, Christelle Delmas, Pierre Delobel, Corine Delsing, Elisa Demonchy, Emmanuelle Denis, Dominique Deplanque, Pieter Depuydt, Mehul Desai, Diane Descamps, Mathilde Desvallées, Santi Dewayanti, Pathik Dhangar, Souleymane Taran Diallo, Alpha Diallo, Sylvain Diamantis, Andrea Dias, André Dias, Priscila Diaz, Rodrigo Diaz, Juan Jose Diaz, Bakary K Dibba, Kévin Didier, Jean-Luc Diehl, Vincent Dinot, Fara Diop, Alphonsine Diouf, Yael Dishon, Cedric Djadda, Félix Djossou, Annemarie B. Docherty, Helen Doherty, Arjen M Dondorp, Christl A. Donnelly, Sean Donohue, Yoann Donohue, Peter Doran, Céline Dorival, Eric D’Ortenzio, Yash Doshi, Phouvieng Douangdala, James Joshua Douglas, Nathalie Dournon, Thomas Drake, Aoife Driscoll, Amiel A. Dror, Murray Dryden, Ibrahim Kwaku Duah, Claudio Duarte Fonseca, Vincent Dubee, François Dubos, Audrey Dubot-Pérès, Alexandre Ducancelle, Toni Duculan, Susanne Dudman, Abhijit Duggal, Paul Dunand, Jake Dunning, Mathilde Duplaix, Emanuele Durante-Mangoni, Lucian Durham III, Bertrand Dussol, Xavier Duval, Anne Margarita Dyrhol-Riise, Sim Choon Ean, Marco Echeverria-Villalobos, Giorgio Economopoulos, Michael Edelstein, Siobhan Egan, Linn Margrete Eggesbø, Khadeja Ehzaz, Carla Eira, Mohammed El Sanharawi, Marwan El Sayed, Mohammed Elabid, Mohamed Bashir Elagili, Mohammad Elbahnasawy, Sohail Elboshra, Brigitte Elharrar, Natalie Elkheir, Jacobien Ellerbroek, Merete Ellingjord-Dale, Hamida ELMagrahi, Rami Elmorsi, Mohammad Muatasm Elmubark, Lauren Eloundou, Philippine Eloy, Abelrahman Elsayed, Basma Elshaikhy, Tarek Elshazly, Wafa Elsokni, Aml Ahmed Eltayeb, Iqbal Elyazar, Zarief Kamel Emad, Hussein Embarek, Isabelle Enderle, Tomoyuki Endo, Gervais Eneli, Chan Chee Eng, Ilka Engelmann, Vincent Enouf, Olivier Epaulard, Haneen Esaadi, Mariano Esperatti, Hélène Esperou, Catarina Espírito Santo, Marina Esposito-Farese, Rachel Essaka, Lorinda Essuman, João Estevão, Manuel Etienne, Hiba Et-taghy, Rachael Evans, Anna Greti Everding, Mirjam Evers, Marc Fabre, Isabelle Fabre, Ismaila Fadera, Asgad Osman Abdalla Fadlalla, Amna Faheem, Mohamed Fahim Elalfy, Arabella Fahy, Hamza Faida, Cameron J. Fairfield, Komal Fareed, Pedro Faria, Ahmed Farooq, Hanan Fateena, Mohamed Fathi, Salem Fatima, Arie Zainul Fatoni, Karine Faure, Raphaël Favory, Mohamed Fayed, Niamh Feely, Marília Andreia Fernandes, Jorge Fernandes, François-Xavier Ferrand, Joana Ferrão, Carlo Ferrari, Mário Ferraz, Sílvia Ferreira, Bernardo Ferreira, Benigno Ferreira, Isabel Ferreira, Nicolas Ferriere, Céline Ficko, Claudia Figueiredo-Mello, Thomas Flament, Tom Fletcher, Aline-Marie Florence, Letizia Lucia Florio, Brigid Flynn, Deirdre Flynn, Federica Fogliazza, Jean Foley, Victor Fomin, Tatiana Fonseca, Patricia Fontela, Karen Forrest, Simon Forsyth, Denise Foster, Giuseppe Foti, Berline Fotso, Mohamed Fouad Abdrabo, Erwan Fourn, Robert A. Fowler, Marianne Fraher, Diego Franch-Llasat, Christophe Fraser, John F. Fraser, Marcela Vieira Freire, Ana Freitas Ribeiro, Caren Friedrich, Ricardo Fritz, Stéphanie Fry, Nora Fuentes, Masahiro Fukuda, Argin G, Valérie Gaborieau, Rostane Gaci, Massimo Gagliardi, Jean-Charles Gagnard, Nathalie Gagné, Amandine Gagneux-Brunon, Abdou Gai, Sérgio Gaião, Linda Gail Skeie, Adham Mohamed Galal Mohamed Ramadan, Phil Gallagher, Elena Gallego Curto, Carrol Gamble, Aysylu Gamirova, Yasmin Gani, Arthur Garan, Rebekha Garcia, Noelia García Barrio, Julia Garcia-Diaz, Esteban Garcia-Gallo, Navya Garimella, Federica Garofalo, Denis Garot, Valérie Garrait, Basanta Gauli, Anatoliy Gavrylov, Alexandre Gaymard, Johannes Gebauer, Eva Geraud, Louis Gerbaud Morlaes, Nuno Germano, Ahmed Noman Ghaleb, Abdulrahman Ghaleb, Malak Ghemmeid, Praveen Kumar Ghisulal, Jade Ghosn, Marco Giani, Carlo Giaquinto, Tristan Gigante, Guillermo Giordano, Michelle Girvan, Valérie Gissot, Jesse Gitaka, Gezy Giwangkancana, Daniel Glikman, Petr Glybochko, Eric Gnall, Geraldine Goco, François Goehringer, Siri Goepel, Jean-Christophe Goffard, Jin Yi Goh, Brigitta Golács, Jonathan Golob, Rui Gomes, Kyle Gomez, Joan Gómez-Junyent, Marie Gominet, Alicia Gonzalez, Patricia Gordon, Yanay Gorelik, Isabelle Gorenne, Laure Goubert, Cécile Goujard, Tiphaine Goulenok, Margarite Grable, Jeronimo Graf, Edward Wilson Grandin, Pascal Granier, Giacomo Grasselli, Lorenzo Grazioli, Christopher A. Green, Courtney Greene, William Greenhalf, Segolène Greffe, Domenico Luca Grieco, Matthew Griffee, Fiona Griffiths, Ioana Grigoras, Albert Groenendijk, Fassou Mathias Grovogui, Heidi Gruner, Yusing Gu, Fabio Guarracino, Jérémie Guedj, Martin Guego, Anne-Marie Guerguerian, Daniela Guerreiro, Romain Guery, Anne Guillaumot, Laurent Guilleminault, Maisa Guimarães de Castro, Thomas Guimard, Marieke Haalboom, Daniel Haber, Ali Hachemi, Abdurrahman Haddud, Nadir Hadri, Wael Hafez, Fakhir Raza Haidri, Fatima Mhd Rida Hajij, Sheeba Hakak, Matthew Hall, Adam Hall, Sophie Halpin, Shaher Hamdan, Abdelhafeez Hamdi, Jawad Hameed, Ansley Hamer, Raph L. Hamers, Rebecca Hamidfar, Bato Hammarström, Naomi Hammond, Terese Hammond, Matly Hanan, Rashan Haniffa, Hayley Hardwick, Samuel Bernard Ekow Harrison, Ewen M. Harrison, Janet Harrison, Alan Hartman, Sulieman Hasan, Mohd Shahnaz Hasan, Mohammad Ali Nabil Hasan, Madiha Hashmi, Junaid Hashmi, Amoni Hassan, Ebtisam Hassanin, Claire Hastie, Muhammad Hayat, Ailbhe Hayes, Leanne Hays, Jan Heerman, Lars Heggelund, Ahmed Helmi, Ross Hendry, Martina Hennessy, Aquiles Rodrigo Henriquez-Trujillo, Maxime Hentzien, Diana Hernandez, Daniel Herr, Andrew Hershey, Astarini Hidayah, Eibhlin Higgins, Samuel Hinton, Hiroaki Hiraiwa, Haider Hirkani, Hikombo Hitoto, Antonia Ho, Yi Bin Ho, Alexandre Hoctin, Isabelle Hoffmann, Wei Han Hoh, Oscar Hoiting, Rebecca Holt, Jan Cato Holter, Peter Horby, Juan Pablo Horcajada, Koji Hoshino, Kota Hoshino, Ikram Houas, Mabrouka Houderi, Catherine L. Hough, Stuart Houltham, Jimmy Ming-Yang Hsu, Jean-Sébastien Hulot, Stella Huo, Abby Hurd, Iqbal Hussain, Mahmood Hussein, Aliae Mohamed Hussein, Fatima Ibrahim, Bashir Ibran, Samreen Ijaz, M. Arfan Ikram, Carlos Cañada Illana, Patrick Imbert, Muhammad Imran Ansari, Rana Imran Sikander, Hugo Inácio, Carmen Infante Dominguez, Yun Sii Ing, Elias Iosifidis, Mariachiara Ippolito, Vera Irawany, Sarah Isgett, Tiago Isidoro, Nadiah Ismail, Margaux Isnard, Mette Stausland Istre, Junji Itai, Asami Ito, Daniel Ivulich, Danielle Jaafar, Salma Jaafoura, Hamza Jaber, Julien Jabot, Clare Jackson, Abubacarr Jagne, Victoria Janes, Waasila Jassat, Stéphane Jaureguiberry, Jeffrey Javidfar, Denise Jaworsky, Florence Jego, Anilawati Mat Jelani, Synne Jenum, Edwin Jesudason, Ruth Jimbo-Sotomayor, Ong Yiaw Joe, Ruth Noemí Jorge García, Silje Bakken Jørgensen, Mark Joseph, Cédric Joseph, Swosti Joshi, Mercé Jourdain, Philippe Jouvet, Hanna Jung, Anna Jung, Dafsah Juzar, Ouifiya Kafif, Florentia Kaguelidou, Neerusha Kaisbain, Thavamany Kaleesvran, Sabrina Kali, Alina Kalicinska, Karl Trygve Kalleberg, Smaragdi Kalomoiri, Muhammad Aisar Ayadi Kamaluddin, Armand Saloun Kamano, Zul Amali Che Kamaruddin, Nadiah Kamarudin, Darshana Hewa Kandamby, Kong Yeow Kang, Darakhshan Kanwal, Dyah Kanyawati, Valentina Kapustina, Mohamed Karghul, Pratap Karpayah, Todd Karsies, Christiana Kartsonaki, Daisuke Kasugai, Kevin Katz, Tatsuya Kawasaki, Christy Kay, Lamees Kayyali, Seán Keating, Pulak Kedia, Aoife Kelly, Niamh Kelly, Yvelynne Kelly, Sadie Kelly, Maeve Kelsey, Sommay Keomany, Maeve Kernan, Younes Kerroumi, Sharma Keshav, Evelyne Kestelyn, Shams Khail, Dina Osman Khair, Sarah Khaled, Imrana Khalid, Antoine Khalil, Quratul Ain Khan, Irfan Khan, Sushil Khanal, Abid Khatak, Krish Kherajani, Michelle E. Kho, Ryan Khoo, Denisa Khoo, Saye Khoo, Muhammad Nasir Khoso, Amin Khuwaja, Yuri Kida, Harrison Kihuga, Peter Kiiza, Beathe Kiland Granerud, Anders Benjamin Kildal, Jae Burm Kim, Antoine Kimmoun, Detlef Kindgen-Milles, Nobuya Kitamura, Stany Kivuruga, Eyrun Floerecke Kjetland, Paul Klenerman, Rob Klont, Gry Kloumann Bekken, Stephen R Knight, Robin Kobbe, Chamira Kodippily, Malte Kohns Vasconcelos, Sabin Koirala, Mamoru Komatsu, Franklina Korkor Abebrese, Volkan Korten, Stephanie Kouba, Karifa Kourouma, Mohamed Lamine Kourouma, Arsène Kpangon, Karolina Krawczyk, Ali Kredan, Sudhir Krishnan, Vinothini Krishnan, Oksana Kruglova, Anneli Krund, Pei Xuan Kuan, Ashok Kumar, Mukesh Kumar, Ganesh Kumar, Deepali Kumar, Dinesh Kuriakose, Ethan Kurtzman, Neurinda Permata Kusumastuti, Demetrios Kutsogiannis, Galyna Kutsyna, Ama Kwakyewaa Bedu-Addo, Sylvie Kwedi, Konstantinos Kyriakoulis, Marie Lachatre, Marie Lacoste, John G. Laffey, Nadhem Lafhej, Marie Lagrange, Fabrice Laine, Olivier Lairez, Sanjay Lakhey, Sulaiman Lakoh, Antonio Lalueza, Marc Lambert, François Lamontagne, Marie Langelot-Richard, Vincent Langlois, Eka Yudha Lantang, Marina Lanza, Cédric Laouénan, Samira Laribi, Delphine Lariviere, Stéphane Lasry, Sakshi Lath, Naveed Latif, Youssef Latifeh, Odile Launay, Didier Laureillard, Yoan Lavie-Badie, Andy Law, Teresa Lawrence, Minh Le, Clément Le Bihan, Cyril Le Bris, Georges Le Falher, Lucie Le Fevre, Quentin Le Hingrat, Marion Le Maréchal, Soizic Le Mestre, Gwenaël Le Moal, Vincent Le Moing, Hervé Le Nagard, Ema Leal, Marta Leal Santos, Biing Horng Lee, Todd C. Lee, Heng Gee Lee, Su Hwan Lee, Yi Lin Lee, Gary Leeming, Bénédicte Lefebvre, Laurent Lefebvre, Benjamin Lefèvre, Sylvie LeGac, Merili-Helen Lehiste, Jean-Daniel Lelievre, François Lellouche, Adrien Lemaignen, Véronique Lemee, Anthony Lemeur, Gretchen Lemmink, Ha Sha Lene, Jenny Lennon, Rafael León, Marc Leone, Tanel Lepik, Quentin Lepiller, François-Xavier Lescure, Olivier Lesens, Mathieu Lesouhaitier, Andrew Letizia, Sophie Letrou, Bruno Levy, Yves Levy, Claire Levy-Marchal, Katarzyna Lewandowska, Gianluigi Li Bassi, Janet Liang, Ali Liaquat, Geoffrey Liegeon, Wei Shen Lim, Kah Chuan Lim, Chantre Lima, Bruno Lina, Lim Lina, Andreas Lind, Maja Katherine Lingad, Guillaume Lingas, Sylvie Lion-Daolio, Samantha Lissauer, Keibun Liu, Marine Livrozet, Patricia Lizotte, Antonio Loforte, Navy Lolong, Leong Chee Loon, Diogo Lopes, Dalia Lopez-Colon, Jose W. López-Revilla, Anthony L. Loschner, Paul Loubet, Bouchra Loufti, Guillame Louis, Silvia Lourenco, Lara Lovelace-Macon, Lee Lee Low, Marije Lowik, Jia Shyi Loy, Jean-Christophe Lucet, Carlos Lumbreras Bermejo, Carlos M. Luna, Olguta Lungu, Miles Lunn, Liem Luong, Nestor Luque, Dominique Luton, Nilar Lwin, Olavi Maasikas, Sarah MacDonald, Sara Machado, Moïse Machado, Gabriel Macheda, Juan Macias Sanchez, Jai Madhok, Guillermo Maestro de la Calle, Jacob Magara, Giuseppe Maglietta, Rachel Maguru, Mustafa Magzoub, Moataz Maher Emara, Mohammed Maher Hadhoud, Rafael Mahieu, Sophie Mahy, Ana Raquel Maia, Lars S. Maier, Oumou Maiga Ascofare, Mylène Maillet, Thomas Maitre, Nimisha Abdul Majeed, Maria Majori, Maximilian Malfertheiner, Nadia Malik, Dayana Malla, Paddy Mallon, Fernando Maltez, Denis Malvy, Patrizia Mammi, Victoria Manda, Jose M. Mandei, Laurent Mandelbrot, Frank Manetta, Julie Mankikian, Edmund Manning, Aldric Manuel, Veronika Maráczi, Ceila Maria Sant’Ana Malaque, Flávio Marino, Samuel Markowicz, Ana Marques, Catherine Marquis, Brian Marsh, Megan Marshal, John Marshall, Celina Turchi Martelli, Dori-Ann Martin, Emily Martin, Guillaume Martin-Blondel, Alessandra Martinelli, F. Eduardo Martinez, Ignacio Martin-Loeches, Martin Martinot, Alejandro Martín-Quiros, Ana Martins, Nuno Martins, João Martins, Caroline Martins Rego, Gennaro Martucci, Olga Martynenko, Eva Miranda Marwali, Marsilla Marzukie, Juan Fernado Masa Jimenez, David Maslove, Sabina Mason, Phillip Mason, Sobia Masood, Fatma Masoud, Moise Massoma, Palmer Masumbe, Mohd Basri Mat Nor, Moshe Matan, Sébastien Matata, Henrique Mateus Fernandes, Meghena Mathew, Christina Mathew, Girish Matjeti, Mathieu Mattei, Laurence Maulin, Juergen May, Jennifer May, Marcel Mayala, Javier Maynar, Mayfong Mayxay, Mohd Zulfakar Mazlan, Thierry Mazzoni, Placide Mbala, Lisa Mc Sweeney, Colin McArthur, Naina McCann, Peter McCanny, Aine McCarthy, Anne McCarthy, Colin McCloskey, Rachael McConnochie, Sherry McDermott, Sarah E. McDonald, Samuel McElwee, Natalie McEvoy, Allison McGeer, Kenneth A. McLean, Paul McNally, Bairbre McNicholas, Edel Meaney, Cécile Mear-Passard, Maggie Mechlin, Ahmed Megdi, Omar Mehkri, Jan Mehrtens, Ferruccio Mele, Luis Melo, Kashif Ali Memon, João João Mendes, Ogechukwu Menkiti, Kusum Menon, France Mentré, Alexander J. Mentzer, Emmanuelle Mercier, Antoine Merckx, Mayka Mergeay-Fabre, Blake Mergler, Tiziana Meschi, António Mesquita, Roberta Meta, Osama Metwally, Agnès Meybeck, Dan Meyer, Alison M. Meynert, Vanina Meysonnier, Mehdi Mezidi, Giuliano Michelagnoli, Isabelle Michelet, Efstathia Mihelis, Vladislav Mihnovit, Duha Milad Abdullah, Jennene Miller, Hugo Miranda-Maldonado, Nor Arisah Misnan, Nouralsabah Mohamed, Nik Nur Eliza Mohamed, Tahira Jamal Mohamed, Alaa Mohamed Ads, Ahmed Reda Mohamed Elsayed Abdelhalim, Libya Mohammed, Shrouk Fawze Mohammed Mostafa, Manahil Omer Abdelrahman Mohammedahmed, Omer Abdullah Mohammedelhassan, Saad A. Moharam, Walaa Mokhtar, Elena Molinos, Brenda Molloy, Mary Mone, Agostinho Monteiro, Claudia Montes, Giorgia Montrucchio, Sarah Moore, Shona C. Moore, Lina Morales Cely, Marwa Morgom, Lucia Moro, Diego Rolando Morocho Tutillo, Ben Morton, Ana Motos, Clara Mouton Perrot, Julien Moyet, Suleiman Haitham Mualla, Caroline Mudara, Mohamed Muftah, Aisha Kalsoom Mufti, Ng Yong Muh, Mo’nes Muhaisen, Dzawani Muhamad, Jackson Muhindo, Daniel Mukadi-Bamuleka, Marithé Mukoka, Jimmy Mullaert, Fredrik Müller, Karl Erik Müller, Daniel Munblit, Muller Mundenga, Syed Muneeb Ali, Nadeem Munir, Laveena Munshi, Aisling Murphy, Patrick Murray, Marlène Murris, Srinivas Murthy, Himed Musaab, Alamin Mustafa, Dana Mustafa, Mus’ab Theeb Mustafa, Carlotta Mutti, Dimitra Melia Myrodia, Farah Nadia Mohd-Hanafiah, Behzad Nadjm, Dave Nagpal, Alex Nagrebetsky, Blanka Nagybányai-Nagy, Herwin Nanda Boudoin, Mangala Narasimhan, Rashid Nasim Khan, Ahmad Nasrallah, Adel Gerges Nassif Metri, Alasdair Nazerali-Maitland, Ebrahim Ndure, Nadège Neant, Coca Necsoi, Nikita Nekliudov, Matthew Nelder, Erni Juwita Nelwan, Raul Neto, Bernardo Neves, Pauline Yeung Ng, Wing Yiu Ng, Anthony Nghi, Jane Ngure, Duc Nguyen, Orna Ni Choileain, Niamh Ni Leathlobhair, Nerissa Niba, Alistair D Nichol, Prompak Nitayavardhana, Stephanie Nonas, Nurul Amani Mohd Noordin, Nurul Faten Izzati Norharizam, Alessandra Notari, Moneer Noureldean, Mahdad Noursadeghi, Adam Nowinski, Saad Nseir, Leonard Numfor, Nurnaningsih Nurnaningsih, Dwi Utomo Nusantara, Elsa Nyamankolly, Anders Benteson Nygaard, Fionnuala O Brien, Annmarie O Callaghan, Annmarie O’Callaghan, Giovanna Occhipinti, Derbrenn OConnor, Max O’Donnell, Ebenezer Oduro-Mensah, Lawrence Ofori-Boadu, Tawnya Ogston, Takayuki Ogura, Tak-Hyuk Oh, Sophie O’Halloran, Katie O’Hearn, Sally-Ann Ohene, Shinichiro Ohshimo, Agnieszka Oldakowska, João Oliveira, Joseph Oliver-Commey, Piero L. Olliaro, Alsarrah Ali Mohammed Omer, Pierre Ondobo, Conar O’Neil, David S. Y. Ong, Jee Yan Ong, Wilna Oosthuyzen, Anne Opavsky, Peter Openshaw, Saijad Orakzai, Claudia Milena Orozco-Chamorro, Andrés Orquera, Mohamed Osama Elsayed Soliman, Javier Osatnik, Linda O’Shea, Miriam O’Sullivan, Siti Zubaidah Othman, Eman Othman, Paul Otiku, Nadia Ouamara, Rachida Ouissa, Christian Owoo, Micheal Owusu, Ama Akyampomaa Owusu-Asare, Clark Owyang, Eric Oziol, Maïder Pagadoy, Justine Pages, Massimo Palmarini, Carlo Palmieri, Giovanna Panarello, Prasan Kumar Panda, Hem Paneru, Lai Hui Pang, Mauro Panigada, Nathalie Pansu, Aurélie Papadopoulos, Paolo Parducci, Edwin Fernando Paredes Oña, Rachael Parke, Melissa Parker, Vieri Parrini, Jérémie Pasquier, Bruno Pastene, Fabian Patauner, Drashti Patel, Mohan Dass Pathmanathan, Luís Patrão, Patricia Patricio, Laura Patrizi, Lisa Patterson, Rajyabardhan Pattnaik, Christelle Paul, Mical Paul, Jorge Paulos, William A. Paxton, Jean-François Payen, Sandra L Peake, Kalaiarasu Peariasamy, Miguel Pedrera Jiménez, Giles J. Peek, Florent Peelman, Nathan Peiffer-Smadja, Vincent Peigne, Mare Pejkovska, Jill Pell, Ithan D. Peltan, Rui Pereira, Daniel Perez, Thomas Perpoint, Antonio Pesenti, Lenina Pessey, Vincent Pestre, Lenka Petrou, Michele Petrovic, Ventzislava Petrov-Sanchez, Frank Olav Pettersen, Gilles Peytavin, Richard Odame Philips, Ooyanong Phonemixay, Soulichanya Phoutthavong, Michael Piagnerelli, Olivier Picone, Maria de Piero, Djura Piersma, Carlos Pimentel, Raquel Pinto, Catarina Pires, Lionel Piroth, Roberta Pisi, Ayodhia Pitaloka, Chiara Piubelli, Riinu Pius, Simone Piva, Laurent Plantier, Hon Shen Png, Julien Poissy, Ryadh Pokeerbux, Maria Pokorska-Spiewak, Sergio Poli, Georgios Pollakis, Diane Ponscarme, Jolanta Popielska, Diego Bastos Porto, Andra-Maris Post, Douwe F. Postma, Pedro Povoa, Diana Póvoas, Jeff Powis, Sofia Prapa, Viladeth Praphasiri, Sébastien Preau, Jean-Charles Preiser, Anton Prinssen, Mark G. Pritchard, Gamage Dona Dilanthi Priyadarshani, Lucia Proença, Sravya Pudota, Bambang Pujo Semedi, Mathew Pulicken, Matteo Puntoni, Peter Puplampu, Luisa Quesada, Vilmaris Quinones-Cardona, Víctor Quirós González, Else Quist-Paulsen, Mohammed Quraishi, Fadi Qutishat, Christian Rabaud, Ebenezer Rabindrarajan, Aldo Rafael, Marie Rafiq, Abdelrahman Ragab, Gabrielle Ragazzo, Mutia Rahardjani, Arslan Rahat Ullah, Rozanah Abd Rahman, Ahmad Kashfi Haji Ab Rahman, Fernando Rainieri, Giri Shan Rajahram, Pratheema Ramachandran, Nagarajan Ramakrishnan, José Ramalho, Kollengode Ramanathan, Ahmad Afiq Ramli, Blandine Rammaert, Grazielle Viana Ramos, Anais Rampello, Muhammad Asim Rana, Rajavardhan Rangappa, Elena Ranza, Christophe Rapp, Thalha Rashan, Aasiyah Rashan, Ghulam Rasheed, Menaldi Rasmin, Indrek Rätsep, Cornelius Rau, Francesco Rausa, Tharmini Ravi, Ali Raza, Andre Real, Stanislas Rebaudet, Sarah Redl, Brenda Reeve, Atta Ur Rehman, Dag Henrik Reikvam, Renato Reis, Jordi Rello, Jonathan Remppis, Hongru Ren, Hanna Renk, Anne-Sophie Resseguier, Matthieu Revest, Oleksa Rewa, Luis Felipe Reyes, Maria Ines Ribeiro, Antonia Ricchiuto, David Richardson, Denise Richardson, Laurent Richier, Siti Nurul Atikah Ahmad Ridzuan, Jordi Riera, Ana L Rios, Asgar Rishu, Patrick Rispal, Karine Risso, Maria Angelica Rivera Nuñez, Doug Robb, Chiara Robba, André Roberto, Charles Roberts, David L. Robertson, Olivier Robineau, Anna Roca, Ferran Roche-Campo, Paola Rodari, Simão Rodeia, Julia Rodriguez Abreu, Bernhard Roessler, Claire Roger, Amanda Rojek, Roberto Roncon-Albuquerque Jr, Mélanie Roriz, Manuel Rosa-Calatrava, Michael Rose, Dorothea Rosenberger, Andrea Rossanese, Matteo Rossetti, Sandra Rossi, Patrick Rossignol, Carine Roy, Benoît Roze, Desy Rusmawatiningtyas, Clark D. Russell, Maeve Ryan, Steffi Ryckaert, Aleksander Rygh Holten, Isabela Saba, Luca Sacchelli, Sairah Sadaf, Musharaf Sadat, Valla Sahraei, Abdurraouf Said, Moumen Said Ellawi, Pranya Sakiyalak, Fodé Bangaly Sako, Moamen Salah, Ali Alaa Salah Eldin Mohamed Abbas, Nawal Salahuddin, Leonardo Salazar, Jodat Saleem, Mohammed Saleh Alyasiri, Talat Ahmed Abu Salem, Gabriele Sales, Charlotte Salmon Gandonniere, Hélène Salvator, Shaden Samardali, Dana Samardali, Yehia Samir Shaaban Aly Orabi, Olivier Sanchez, Emely Sanchez, Xavier Sánchez Choez, Kizy Sanchez de Oliveira, Angel Sanchez-Miralles, Zulfiqar Sandhu, Gyan Sandhu, Pierre-François Sandrine, Oana Săndulescu, Marlene Santos, Shirley Sarfo-Mensah, Bruno Sarmento Banheiro, Benjamine Sarton, Ankana Satya, Sree Satyapriya, Rumaisah Satyawati, Parthena Savvidou, Yen Tsen Saw, Islam Sayed, Justin Schaffer, Tjard Schermer, Arnaud Scherpereel, Marion Schneider, János Schnur, Michael Schwameis, Gary Schwartz, Janet T. Scott, Nicholas Sedillot, Tamara Seitz, Jaganathan Selvanayagam, Mageswari Selvarajoo, Malcolm G. Semple, Rasidah Bt Senian, Eric Senneville, Claudia Sepulveda, Tânia Sequeira, Filipa Sequeira, Ary Serpa Neto, Pablo Serrano Balazote, Ellen Shadowitz, Syamin Asyraf Shahidan, Hamza Shahla, Laila Shalabi, Haitam Shames, Anuraj Shankar, Shaikh Sharjeel, Pratima Sharma, Catherine A. Shaw, Victoria Shaw, Wejdan Ahmed Shawlan, Ahmed Shazly, John Robert Sheenan, Dr. Rajesh Mohan Shetty, Rohan Shetty, Nisreen Shiban, Mohiuddin Shiekh, Takuya Shiga, Nobuaki Shime, Naoki Shimizu, Hiroaki Shimizu, Keiki Shimizu, Sally Shrapnel, Shubha Kalyan Shrestha, Pramesh Sundar Shrestha, Hoi Ping Shum, Nassima Si Mohammed, Ng Yong Siang, Moses Siaw-Frimpong, Jeanne Sibiude, Bountoy Sibounheuang, Nidhal Siddig, Atif Siddiqui, Maqsood Ahmed Siddiqui, Louise Sigfrid, Fatoumata Sillah, Piret Sillaots, Catarina Silva, Maria Joao Silva, Rogério Silva, Benedict Sim Lim Heng, Wai Ching Sin, Dario Sinatti, Girish Sindhwani, Punam Singh, Mahendra Singh, Pompini Agustina Sitompul, Karisha Sivam, Vegard Skogen, Sue Smith, Benjamin Smood, Coilin Smyth, Dominic So, Tze Vee Soh, Lene Bergendal Solberg, Tom Solomon, Joshua Solomon, Emily Somers, Agnès Sommet, Tae Song, Rima Song, Myung Jin Song, Jack Song Chia, Arne Søraas, Albert Sotto, Edouard Soum, Marta Sousa, Ana Chora Sousa, Maria Sousa Uva, Vicente Souza-Dantas, Mamadou Saliou Sow, Alexandra Sperry, Elisabetta Spinuzza, Ekaterina Spiridonova, B. P. Sanka Ruwan Sri Darshana, Shiranee Sriskandan, Sarah Stabler, Thomas Staudinger, Stephanie-Susanne Stecher, Trude Steinsvik, Ymkje Stienstra, Birgitte Stiksrud, Eva Stolz, Amy Stone, Zachary Stotz, Anca Streinu-Cercel, Adrian Streinu-Cercel, Geoff Strong, Ami Stuart, David Stuart, Decy Subekti, Gabriel Suen, Jacky Y. Suen, Prasanth Sukumar, Asfia Sultana, Charlotte Summers, Dubravka Supic, Deepashankari Suppiah, Magdalena Surovcová, Atie Suwarti, Andrey Svistunov, Sarah Syahrin, Augustina Sylverken, Konstantinos Syrigos, Jaques Sztajnbok, Konstanty Szuldrzynski, Shirin Tabrizi, Fabio S. Taccone, Shahdattul Mawarni Taib, Ewa Talarek, Sara Taleb, Cheikh Talla, Jelmer Talsma, Renaud Tamisier, Maria Lawrensia Tampubolon, Yan Chyi Tan, Le Van Tan, Kim Keat Tan, Taku Tanaka, Clarice Tanaka, Hiroyuki Tanaka, Hayato Taniguchi, Huda Taqdees, Arshad Taqi, Coralie Tardivon, Yousef Tarek Kamal Mostafa, Ali Tarhabat, Pierre Tattevin, M Azhari Taufik, Hassan Tawfik, Tze Yuan Tee, João Teixeira, Sofia Tejada, Marie-Capucine Tellier, Sze Kye Teoh, Vanessa Teotonio, François Téoulé, Olivier Terrier, Nicolas Terzi, Hubert Tessier-Grenier, Adrian Tey, Alif Adlan Mohd Thabit, Anand Thakur, Zhang Duan Tham, Suvintheran Thangavelu, Samar Tharwat, Elmi Theron, Vincent Thibault, Simon-Djamel Thiberville, Benoît Thill, Jananee Thirumanickam, Shaun Thompson, Niamh Thompson, David Thomson, Emma C. Thomson, Mathew Thorpe, Surain Raaj Thanga Thurai, Duong Bich Thuy, Ryan S. Thwaites, Andrea Ticinesi, Paul Tierney, Vadim Tieroshyn, Peter S Timashev, Jean-François Timsit, Noémie Tissot, Jordan Zhien Yang Toh, Maria Toki, Kristian Tonby, Sia Loong Tonnii, Marta Torre, Antoni Torres, Margarida Torres, Rosario Maria Torres Santos-Olmo, Hernando Torres-Zevallos, Aboubacar Tounkara, Michael Towers, Fodé Amara Traoré, Tony Trapani, Anastasia Trefilova, Cécile Tromeur, Ioannis Trontzas, Jeanne Truong, Christelle Tual, Sarah Tubiana, Helen Tuite, Alexis F. Turgeon, Lance C.W. Turtle, Anders Tveita, Pawel Twardowski, PG Ishara Udayanga, Andrew Udy, Roman Ullrich, Alberto Uribe, Asad Usman, Effua Usuf, Timothy M. Uyeki, Michel Vaillant, Patemo Vainitoba, Cristinava Vajdovics, Piero Valentini, Luís Val-Flores, Ana Luiza Valle, Ilaria Valzano, Stijn Van de Velde, Marcel van den Berge, Machteld van der Feltz, Job van der Palen, Paul van der Valk, Nicky Van Der Vekens, Peter Van der Voort, Sylvie Van Der Werf, Marlice van Dyk, Laura van Gulik, Jarne Van Hattem, Carolien van Netten, Frank van Someren Gréve, Gitte Van Twillert, Ilonka van Veen, Hugo Van Willigen, Noémie Vanel, Henk Vanoverschelde, Michael Varrone, Shoban Raj Vasudayan, Charline Vauchy, Pavan Kumar Vecham, Shaminee Veeran, Aurélie Veislinger, Sara Ventura, Annelies Verbon, Hervé Viala, José Ernesto Vidal, César Vieira, Deepak Vijayan, Judit Villar, Pierre-Marc Villeneuve, Andrea Villoldo, Nguyen Van Vinh Chau, Gayatri Vishwanathan, Hannah Visser, Manivanh Vongsouvath, Harald Vonkeman, Fanny Vuotto, Noor Hidayu Wahab, Suhaila Abdul Wahab, Nadirah Abdul Wahid, Louise Wain, Marina Wainstein, Laura Walsh, Wan Fadzlina Wan Muhd Shukeri, Chih-Hsien Wang, Steve Webb, Jia Wei, Katharina Weil, Tan Pei Wen, Hassi Wesam, Sanne Wesselius, T. Eoin West, Murray Wham, Bryan Whelan, Nicole White, Paul Henri Wicky, Aurélie Wiedemann, Surya Otto Wijaya, Keith Wille, Sue Willems, Patricia J Williams, Bailey Williams, Virginie Williams, David Williamson, Evert-Jan Wils, Jessica Wittman, Xin Ci Wong, Calvin Wong, Teck Fung Wong, Yew Sing Wong, Natalie Wright, Lim Saio Xian, Ioannis Xynogalas, Sophie Yacoub, Siti Rohani Binti Mohd Yakop, Masaki Yamazaki, Elizabeth Yarad, Yazdan Yazdanpanah, Nicholas Yee Liang Hing, Abdelrahman Yehia Mahmoud Abdelaal, Cécile Yelnik, Chian Hui Yeoh, Touxiong Yiaye, Toshiki Yokoyama, Hodane Yonis, Obada Yousif, Saptadi Yuliarto, Akram Zaaqoq, Marion Zabbe, Gustavo E. Zabert, Masliza Zahid, Nor Zaila Binti Zaidan, Maria Zambon, Miguel Zambrano, Mostafa Zanaty, Alberto Zanella, Konrad Zawadka, Nurul Zaynah, Hiba Zayyad, Alexander Zoufaly, David Zucman, CCP UK, Mazankowski Heart Institute, PHOSP Collaborative Group, and The Western Australian COVID-19 Research Response.
