# Supplementary material for: Optimising Clinical Epidemiology in Disease Outbreaks: Analysis of ISARIC-WHO COVID-19 Case Report Form Utilisation
Source: Epidemiologia (Basel). 2024 Aug 30;5(3):557–80. doi: 10.3390/epidemiologia5030039 (PMC11417906; doi:10.3390/epidemiologia5030039)
Supplement: Supplementary file 1 [file epidemiologia-05-00039-s001.zip › ISARIC-WHO COVID-19 CRF 08DEC21.pdf]

# COVID-19 CORE CASE REPORT FORM

## ACUTE RESPIRATORY INFECTION CLINICAL CHARACTERISATION DATA TOOL

***DESIGN OF THIS CASE REPORT FORM (CRF)***

This CRF is set up in modules to be used for recording data on the ISARIC COVID-19 Core Database or for independent studies.

**Module 1 and Module 2** complete on the first day of presentation/admission or on first day of COVID-19 assessment.
**Module 2** also complete on first day of admission to ICU or high dependency unit, or if receiving critical care in any ward, and on any days that research specific samples are taken. In addition, complete daily if of interest for local, specific analysis. Continue to follow-up patients who transfer between wards.
**Module 3** (Outcome) complete at discharge or death

**GENERAL GUIDANCE**

- The CRF is designed to collect data obtained through examination, interview and review of hospital notes. Data may be collected prospectively or retrospectively if the patient is enrolled after the admission date.
- For more detailed guidance on how to complete these forms, please refer to the CRF Completion Guideline
- Participant Identification Numbers consist of a 3 or 5 digit site code and a 4 digit participant number. You can obtain a site code and register on the data management system by contacting *ncov@isaric.org*. Participant numbers should be assigned sequentially for each site beginning with 0001. In the case of a single site recruiting participants on different wards, or where it is otherwise difficult to assign sequential numbers, it is acceptable to assign numbers in blocks or incorporate alpha characters. E.g. Ward X will assign numbers from 0001 or A001 onwards and Ward Y will assign numbers from 5001 or B001 onwards. Enter the Participant Identification Number at the top of every page.
- Printed paper CRFs may be used for later transfer of the data onto the electronic database.
- For participants who return for re-admission to the same site, **start a new form with a different Participant Identification Number**. Please check "YES-admitted previously to this facility" in the RE-ADMISSION section. Enter as 2 separate entries in the electronic database.
- For participants who transfer between two sites that are both collecting data on this form, it is preferred to have the data entered by a single site as a single admission, under the same Participant Identification Number. When this is not possible, the first site should record "Transfer to other facility" as an OUTCOME, and the second site should start a new form with a new patient number and indicate "YES-transferred from other facility" RE-ADMISSION.
- Complete every line of every section, except where the instructions say to skip a section based on a response.
- Selections with circles (**O**) are single selection answers (choose one answer only). Selections with square boxes (☐) are multiple selection answers (choose as many answers as are applicable).
- Mark 'Not done' for any results of laboratory values that are not available, not applicable or unknown.
- Avoid recording data outside of the dedicated areas. Sections are available for recording additional information.
- If using paper CRFs, we recommend writing clearly in ink, using BLOCK-CAPITAL LETTERS.
- Place an (X) when you choose the corresponding answer. To make corrections, strike through (-------) the data you wish to delete and write the correct data above it. Please initial and date all corrections.
- Please keep all of the sheets for a single participant together e.g. with a staple or participant-unique folder.
- Please transfer all paper CRF data to the electronic database. All paper CRFs needs to be stored locally, do not send any forms to us. Data are accepted only via secure electronic database.
- Please enter data on the electronic data capture system at https://ncov.medsci.ox.ac.uk/. If your site would like to collect data independently, we are happy to support the establishment of locally hosted databases.
- Please contact us at *ncov@isaric.org* if you need help with databases, if you have comments and to let us know that you are using the forms.


Adapted from SPRINT SARI CRF by ISARIC. Licensed under a Creative Commons Attribution-ShareAlike 4.0 International License by ISARIC on behalf of Oxford University.

PARTICIPANT IDENTIFICATION #: [___][___][___][___][___]--- [___][___][___][___]

# MODULE 1: PRESENTATION/ADMISSION CASE REPORT FORM

## CLINICAL INCLUSION CRITERIA

**Suspected or confirmed novel coronavirus (COVID-19) infection:** ○YES ○NO

**Is COVID-19 the reason for hospital admission?**

○Yes, COVID-19 is the reason for hospital admission

○No, the patient is admitted to hospital for a reason other than COVID-19

## DEMOGRAPHICS

**Clinical centre name:** ________________________ **Country:** ____________________

**Enrolment date /first COVID-19 assessment date:** [_D_][_D_]/[_M_][_M_]/[_2_][_0_][_Y_][_Y_]

**Ethnic group** *(check all that apply):* ☐Arab ☐Black ☐East Asian ☐South Asian ☐ West Asian ☐Latin American ☐White ☐Aboriginal/First Nations ☐Other: ____________________ ○Unknown

**Employed as a Healthcare Worker?** ○YES ○NO ○Unknown **Employed in a microbiology laboratory?** ○YES ○NO ○Unknown

**Sex at Birth:** ○Male ○Female ○Not specified/Unknown **Age** [___][___][___]years OR [___][___]months

**Pregnant?** ○YES ○NO ○Unknown **If YES: Gestational weeks assessment:** [___][___] weeks


# MODULE 1: PRESENTATION/ADMISSION CASE REPORT FORM


## ONSET & ADMISSION

**Onset date of first/earliest symptom:** [_D_][_D_]/[_M_][_M_]/[_2_][_0_][_Y_][_Y_]

**Most recent presentation/admission date at this facility:** [_D_][_D_]/[_M_][_M_]/[_2_][_0_][_Y_][_Y_]

## SIGNS AND SYMPTOMS AT HOSPITAL ADMISSION *(first available data at presentation/admission – within 24 hours)*

**Temperature:** [____][____][____].[____]○°C *or* ○°F

**HR:** [____][____][____]beats/minute **RR:** [____][____]breaths/minute

**Systolic BP:** [___][___][___]mmHg **Diastolic BP:** [____][____][____]mmHg

**Oxygen saturation:** [___][___][___]% **On:** ○Room air ○Oxygen therapy ○Unknown

**Sternal capillary refill time >2sec.** ○YES ○NO ○Unknown **Height:** [___][___][___]cm **Weight:** [___][___][___]kg

| SIGNS AND SYMPTOMS ON ADMISSION *(Unk = Unknown)* | | | |
|---|---|---|---|
| History of fever | ○YES ○NO ○Unk | Fatigue / Malaise | ○YES ○NO ○Unk |
| Cough ○YES - non-productive | ○YES - productive | Anorexia | ○YES ○NO ○Unk |
| ○YES - with haemoptysis | ○NO ○Unk | Altered consciousness/confusion | ○YES ○NO ○Unk |
| Sore throat | ○YES ○NO ○Unk | Muscle aches (myalgia) | ○YES ○NO ○Unk |
| Runny nose (rhinorrhoea) | ○YES ○NO ○Unk | Joint pain (arthralgia) | ○YES ○NO ○Unk |




Adapted from SPRINT SARI CRF by ISARIC. Licensed under a Creative Commons Attribution-ShareAlike 4.0 International License by ISARIC on behalf of Oxford University.

| | | | |
|---|---|---|---|
| Wheezing | OYES ONO OUnk | Inability to walk | OYES ONO OUnk |
| Shortness of breath | OYES ONO OUnk | Abdominal pain | OYES ONO OUnk |
| Lower chest wall indrawing | OYES ONO OUnk | Diarrhoea | OYES ONO OUnk |
| Chest pain | OYES ONO OUnk | Vomiting / Nausea | OYES ONO OUnk |
| Conjunctivitis | OYES ONO OUnk | Skin rash | OYES ONO OUnk |
| Lymphadenopathy | OYES ONO OUnk | Bleeding (Haemorrhage) | OYES ONO OUnk |
| Headache | OYES ONO OUnk | If YES, specify site(s):______________________________ | |
| Loss of smell (Anosmia) | OYES ONO OUnk | Other symptom(s) | OYES ONO OUnk |
| Loss of taste (Ageusia) | OYES ONO OUnk | If YES, specify:______________________________ | |
| Seizures | OYES ONO OUnk | | |

# MODULE 1: PRESENTATION/ADMISSION CASE REPORT FORM

| **PRE-ADMISSION MEDICATION** *(taken within 14 days prior to admission/presentation at healthcare facility)* | |
|---|---|
| Steroids | OYES ONO OUnk If YES, OOral OInhaled OUnk |
| Other immunosuppressant agents (not oral steroids) | OYES ONO OUnk |
| Antibiotics | OYES ONO OUnk If YES, agent(s):______________________________ |
| Antivirals | OYES ONO OUnk If YES, agent(s):______________________________ |
| Other targeted COVID-19 Medications | OYES ONO OUnk If YES, agent(s):______________________________ |

| **CO-MORBIDITIES AND RISK FACTORS** *(existing prior to admission and ongoing)* | | | |
|---|---|---|---|
| Chronic cardiac disease *(not hypertension)* | OYES ONO OUnk | Chronic hematologic disease | OYES ONO OUnk |
| Hypertension | OYES ONO OUnk | AIDS / HIV OYES-on ART OYES-not on ART ONO OUnk<br>If YES, most recent CD4 count:<br>O< 200 O200-< 500 O≥ 500 cells/uL OUnk | |
| Chronic pulmonary disease *(not asthma)* | OYES ONO OUnk | Diabetes Mellitus OYES-Type 1 OYES -Type 2 OYES -Gestational ONO OUnk<br>If YES, HbA1C results (within last 6 months) :_________<br>Units: Ommol/mol Ommol/L O% | |
| Asthma *(physician diagnosed)* | OYES ONO OUnk | Rheumatologic disorder | OYES ONO OUnk |
| Chronic kidney disease | OYES ONO OUnk | Dementia | OYES ONO OUnk |
| Obesity *(as defined by clinical staff)* | OYES ONO OUnk | Tuberculosis | OYES ONO OUnk |
| Moderate or severe liver disease | OYES ONO OUnk | Malnutrition | OYES ONO OUnk |
| Mild liver disease | OYES ONO OUnk | Smoking OYES ONever smoked OFormer smoker OUnk | |
| Asplenia | OYES ONO OUnk | Other relevant risk factor(s) | OYES ONO OUnk |
| Chronic neurological disorder | OYES ONO OUnk | If YES, specify: | |
| Malignant neoplasm | OYES ONO OUnk | | |




Adapted from SPRINT SARI CRF by ISARIC. Licensed under a Creative Commons Attribution-ShareAlike 4.0 International License by ISARIC on behalf of Oxford University.

# MODULE 2: CASE REPORT FORM ON ADMISSION, CRITICAL CARE, RESEARCH SAMPLING

*Complete on the day of admission or first COVID-19 investigation, and on the first day of ICU admission (if different from day of admission). In addition, complete for days when biochemical results are available.*

## SIGNS AND SYMPTOMS *(Record the worst value between 00:00 to 24:00 on day of assessment)(worst=furthest from normal range)*

**DATE OF ASSESSMENT** *(DD/MM/YYYY)*: [_D_][_D_]/[_M_][_M_]/[_2_][_0_][_Y_][_Y_]

**Highest temperature:** [___][___][___].[___] O°C *or* O°F **HR:** [___][___][___]beats/minute **RR:** [___][___][___]breaths/minute

**Systolic BP:** [___][___][___]mmHg **Diastolic BP:** [___][___][___]mmHg

**Oxygen saturation $SaO_2$** [___][___][___]%

**Any supplemental oxygen:** OYES ONO OUnknown If yes,

**$FiO_2$ *(0.21-1.0)*** [___].[___][___] or [___][___] % or [___][___]L/min (Highest L/min)

**$PaO_2$** *(at time nearest to the $FiO_2$ recorded at top of page)* [___][___][___]OkPa *or* OmmHg ONot done

**$PaO_2$ sample type:** OArterial OCapillary OVenous OUnknown

**From same blood gas record as $PaO_2$:**

**$PCO_2$** ____________OkPa *or* OmmHg | **pH** _____________ | **$HCO_3$-** ___________mEq/L | **Base excess** __________ mmol/L

**Sternal capillary refill time >2seconds** **OYES ONO OUnknown**

**AVPU: Alert [___] Verbal[___] Pain [___] Unresponsive [___]** **Glasgow Coma Score (GCS / 15) [___][___]**

**Richmond Agitation-Sedation Scale (RASS) [___]**

**Mean Arterial Blood Pressure** [___][___][___]mmHg OUnknown

**Urine flow rate** [___][___][___][___][___]mL/24 hours O Check if estimated OUnknown

## Is the patient currently receiving, or has received *(between 00:00 to 24:00 on day of assessment)*

**Current admission to ICU/ITU/IMC/HDU?** OYES ONO OUnknown

**High-flow nasal cannula oxygen therapy?** OYES ONO OUnknown

**Non-invasive ventilation *(Any)*?** OYES ONO OUnknown **If YES:** OBIPAP OCPAP OOther OUnknown

**Invasive ventilation?** OYES ONO OUnknown

**Prone positioning?** OYES ONO OUnknown If yes, Oduring invasive ventilation Owhilst self-ventilating OUnknown

**Inhaled Nitric Oxide?** OYES ONO OUnknown

**Tracheostomy inserted?** OYES ONO OUnknown

**Extra corporeal life support (ECLS/ ECMO)?** OYES ONO OUnknown **If YES:** OVV OAV OCentral OUnknown

**Renal replacement therapy (RRT) or dialysis?** OYES ONO OUnknown

**Any vasopressor/inotropic support?** OYES ONO OUnknown *(if NO, select NO for the next 3 questions)*

**Dopamine <5µg/kg/min OR Dobutamine OR milrinone OR levosimendan:** OYES ONO

**Dopamine 5-15µg/kg/min OR Epinephrine/Norepinephrine < 0.1µg/kg/min OR vasopressin OR phenylephrine**: OYES ONO

**Dopamine >15µg/k/min OR Epinephrine/Norepinephrine > 0.1µg/kg/min**: OYES ONO

**Neuromuscular blocking agents?** OYES ONO OUnknown

**Other intervention(s) or procedure(s)?** OYES ONO OUnknown If YES, Specify: _______

_______________________________________________





Adapted from SPRINT SARI CRF by ISARIC. Licensed under a Creative Commons Attribution-ShareAlike 4.0 International License by ISARIC on behalf of Oxford University.

PARTICIPANT IDENTIFICATION #: [___][___][___][___][___]--- [___][___][___][___]

# MODULE 2: CASE REPORT FORM ON ADMISSION, CRITICAL CARE, RESEARCH SAMPLING

*Complete on the day of admission or first COVID-19 investigation, and on the first day of ICU admission (if different from day of admission). In addition, complete for days when biochemical results are available.*

| **LABORATORY RESULTS (on admission, on any admission to ICU, then daily) – complete every line** | | | | | |
|---|---|---|---|---|---|
| **DATE OF ASSESSMENT** *(DD/MM/YYYY)*: [_D_][_D_]/[_M_][_M_]/[_2_][_0_][_Y_][_Y_] | | | | | |
| **LABORATORY RESULTS** (**record units if different from those listed*)<br>Record the worst value between 00:00 to 24:00 on day of assessment (if Not Available write 'N/A') | | | | | |
| **Parameter** | **Value*** | **Not done** | **Parameter** | **Value*** | **Not done** |
| Haemoglobin (g/L) | | ○ | Urea (BUN) (mmol/L) | | ○ |
| WBC count (x10$^9$/L) | | ○ | Lactate (mmol/L) | | ○ |
| Lymphocyte count (10$^9$/L) | | ○ | Creatinine (µmol/L) | | ○ |
| Neutrophil count (10$^9$/L) | | ○ | Sodium (mmol/L) | | ○ |
| Haematocrit (%) | | ○ | Potassium (mmol/L) | | ○ |
| Platelets (x10$^9$/L) | | ○ | Procalcitonin (ng/mL) | | ○ |
| APTT (seconds)) | | ○ | CRP (mg/L) | | ○ |
| APTR | | ○ | LDH (U/L) | | ○ |
| PT (seconds) | | ○ | Creatine kinase (U/L) | | ○ |
| INR | | ○ | Troponin I (ng/mL) | | ○ |
| ALT/SGPT (U/L) | | ○ | D-dimer (mg/L) | | ○ |
| Total bilirubin (µmol/L) | | ○ | Ferritin (ng/mL) | | ○ |
| AST/SGOT (U/L) | | ○ | IL-6 (pg/mL) | | ○ |
| Glucose (mmol/L) | | ○ | Fibrinogen (mg/dl) | | ○ |

# MODULE 3: OUTCOME CASE REPORT FORM

**TREATMENT: At ANY time during hospitalisation, did the patient receive/undergo:**

**Any Oxygen therapy?** ○YES ○NO ○Unknown **If YES, total duration:** _________days ○Unknown

**Maximum $O_2$ flow volume:** ○<2 L/min ○2-5 L/min ○6-10 L/min ○11-15 L/min ○>15 L/min

**Non-invasive ventilation?** *(Any)* ○YES ○NO ○Unknown **If YES, total duration:** _________days ○Unknown

**Invasive ventilation?** *(Any)* ○YES ○NO ○Unknown **If YES, total duration:** _________days ○Unknown

**High flow nasal oxygen** ○YES ○NO ○Unknown **If YES, total duration:** _________days ○Unknown

**Prone Positioning?** ○YES ○NO ○Unknown

**Inhaled Nitric Oxide?** ○YES ○NO ○Unknown

**Tracheostomy inserted?** ○YES ○NO ○Unknown

**Extracorporeal support (ECMO)?** ○YES ○NO ○Unknown **If YES, total duration:** _________days ○Unknown

**Renal replacement therapy (RRT) or dialysis?** ○YES ○NO ○Unknown

**Inotropes/vasopressors?** ○YES ○NO ○Unknown

**ICU or High Dependency Unit admission?** ○YES ○NO ○Unknown **If YES, total duration:** _________days ○Unknown

**If YES, date of ICU admission:** [_D_][_D_]/[_M_][_M_]/[_2_][_0_][_Y_][_Y_] ○Unknown

**date of ICU discharge:** [_D_][_D_]/[_M_][_M_]/[_2_][_0_][_Y_][_Y_] ○Unknown



Adapted from SPRINT SARI CRF by ISARIC. Licensed under a Creative Commons Attribution-ShareAlike 4.0 International License by ISARIC on behalf of Oxford University.

# MODULE 3: OUTCOME CASE REPORT FORM

| **COMPLICATIONS: At any time during hospitalisation did the patient experience:** *(Unk = Unknown)* | | | |
|---|---|---|---|
| Viral pneumonia/pneumonitis | OYES ONO OUnk | Meningitis / Encephalitis | OYES ONO OUnk |
| Bacterial pneumonia | OYES ONO OUnk | Bacteremia | OYES ONO OUnk |
| Acute Respiratory Distress Syndrome | OYES ONO OUnk | Coagulation disorder / DIC | OYES ONO OUnk |
| Pneumothorax | OYES ONO OUnk | Pulmonary Embolism | OYES ONO OUnk |
| Pleural effusion | OYES ONO OUnk | Deep Vein Thrombosis | OYES ONO OUnk |
| Cryptogenic organizing pneumonia (COP) | OYES ONO OUnk | Other thromboembolism (not PE or DVT) | OYES ONO OUnk |
| Bronchiolitis | OYES ONO OUnk | Anemia | OYES ONO OUnk |
| Cardiac arrest | OYES ONO OUnk | Rhabdomyolysis / Myositis | OYES ONO OUnk |
| Myocardial infarction | OYES ONO OUnk | Acute renal injury/ Acute renal failure | OYES ONO OUnk |
| Cardiac ischaemia | OYES ONO OUnk | Gastrointestinal haemorrhage | OYES ONO OUnk |
| Cardiac arrhythmia | OYES ONO OUnk | Pancreatitis | OYES ONO OUnk |
| Myocarditis / Pericarditis | OYES ONO OUnk | Liver dysfunction | OYES ONO OUnk |
| Endocarditis | OYES ONO OUnk | Hyperglycemia | OYES ONO OUnk |
| Cardiomyopathy | OYES ONO OUnk | Hypoglycemia | OYES ONO OUnk |
| Congestive heart failure | OYES ONO OUnk | Other | OYES ONO OUnk |
| Seizure | OYES ONO OUnk | If YES, specify:_______________________________ | |
| Stroke / Cerebrovascular accident | OYES ONO OUnk | | |

## DIAGNOSTICS

**Section 1: RESPIRATORY VIRUS PCR TESTING**

**SARS-CoV-2 (COVID-19):** OPositive ONegative ONot done OUnknown

**Was other pathogen testing done during this illness episode?** OYES *(complete section)* ONO OUnknown

**Influenza :** OPositive ONegative ONot done OUnknown

**If Positive:** OA-not typed OA/H3N2 OA/H1N1pdm09 OA/H7N9 OA/H5N1 OB OOther:_____________________ ..OUnk

**Respiratory Syncytial Virus (RSV):** OPositive ONegative ONot done OUnknown

**Adenovirus:** OPositive ONegative ONot done OUnknown

**Section 2: BACTERIAL TESTING**

**Bacteria:** OPositive ONegative ONot done **If Positive, specify**: ___________________________________ OUnknown

**Other pathogen/s detected:** OYES ONO OUnknown **If YES, specify all:**___________________________________OUnknown

**Section 3: RADIOLOGY**

**Clinical pneumonia diagnosed?** OYES ONO OUnknown

**Chest X-Ray performed?** OYES ONO OUnknown **If Yes: Were infiltrates present?** OYES ONO OUnknown

**CT performed?** OYES ONO OUnknown **If Yes: Were infiltrates present?** OYES ONO OUnknown


Adapted from SPRINT SARI CRF by ISARIC. Licensed under a Creative Commons Attribution-ShareAlike 4.0 International License by ISARIC on behalf of Oxford University.

# MODULE 3: OUTCOME CASE REPORT FORM

## DIAGNOSTICS *continued*

### Section 4: PATHOGEN TESTING DETAILS

| Collection Date *(DD/MM/YYYY)* | Biospecimen Type | Laboratory test Method | Result | Pathogen Tested/Detected |
|---|---|---|---|---|
| _D_ _D_ / _M_ _M_ /20_Y_ _Y_ | ○Nasal/NP swab ○Throat swab<br>○Combined nasal/NP+throat swab<br>○Sputum ○BAL ○ETA ○Urine<br>○Feces/rectal swab ○Blood<br>○Other, *Specify:* ________ | ○PCR<br>○Culture<br>○Other, *Specify:*<br>________ | ○Positive<br>○Negative<br>○Unknown | ________ |
| _D_ _D_ / _M_ _M_ /20_Y_ _Y_ | ○Nasal/NP swab ○Throat swab<br>○Combined nasal/NP+throat swab<br>○Sputum ○BAL ○ETA ○Urine<br>○Feces/rectal swab ○Blood<br>○Other, *Specify:* ________ | ○PCR<br>○Culture<br>○Other, *Specify:*<br>________ | ○Positive<br>○Negative<br>○Unknown | ________ |
| _D_ _D_ / _M_ _M_ /20_Y_ _Y_ | ○Nasal/NP swab ○Throat swab<br>○Combined nasal/NP+throat swab<br>○Sputum ○BAL ○ETA ○Urine<br>○Feces/rectal swab ○Blood<br>○Other, *Specify:* ________ | ○PCR<br>○Culture<br>○Other, *Specify:*<br>________ | ○Positive<br>○Negative<br>○Unknown | ________ |
| _D_ _D_ / _M_ _M_ /20_Y_ _Y_ | ○Nasal/NP swab ○Throat swab<br>○Combined nasal/NP+throat swab<br>○Sputum ○BAL ○ETA ○Urine<br>○Faeces/rectal swab ○Blood<br>○Other, *Specify:* ________ | ○PCR<br>○Culture<br>○Other, *Specify:*<br>________ | ○Positive<br>○Negative<br>○Unknown | ________ |

## MEDICATION: While hospitalised or at discharge, were any of the following administered? *(Unk=Unknown)*

**ANTIVIRAL OR COVID-19 TARGETED AGENT?** ○YES ○NO ○Unknown **If YES, specify (all) :**

☐ **Ribavirin** **Date commenced**[_D_][_D_]/[_M_][_M_]/[_2_][_0_][_Y_][_Y_] ○Unk **Duration:** ________ days ○Unk

☐ **Lopinavir/Ritonavir** **Date commenced** [_D_][_D_]/[_M_][_M_]/[_2_][_0_][_Y_][_Y_] ○Unk **Duration:** ________ days ○Unk

☐ **Remdesivir (Veklury)** **Date commenced** [_D_][_D_]/[_M_][_M_]/[_2_][_0_][_Y_][_Y_] ○Unk **Duration:** ________ days ○Unk

☐ **Interferon alpha** **Date commenced** [_D_][_D_]/[_M_][_M_]/[_2_][_0_][_Y_][_Y_] ○Unk **Duration:** ________ days ○Unk

☐ **Interferon beta** **Date commenced** [_D_][_D_]/[_M_][_M_]/[_2_][_0_][_Y_][_Y_] ○Unk **Duration:** ________ days ○Unk

☐ **Chloroquine/hydroxychloroquine:**

**Date commenced** [_D_][_D_]/[_M_][_M_]/[_2_][_0_][_Y_][_Y_] ○Unk... **Duration:** ________days ○Unk

☐ **Interleukin-6 (IL-6) inhibitor** **IF YES which:** ☐ Tocilizumab ☐ Sarilumab ☐ Other IL-6 inhibitor________________________ ○Unk

**Date commenced** [_D_][_D_]/[_M_][_M_]/[_2_][_0_][_Y_][_Y_] ○Unk... **Duration:** ________days ○Unk

☐ **Convalescent plasma** **Date commenced** [_D_][_D_]/[_M_][_M_]/[_2_][_0_][_Y_][_Y_] ○Unk **Duration:** ________days ○Unk

☐ **Anti-influenza anti-viral** **IF YES which:** ☐**Oseltamivir (Tamiflu®)** ☐ **Zanamivir** ○Unk

**Date commenced** [_D_][_D_]/[_M_][_M_]/[_2_][_0_][_Y_][_Y_] ○Unk... **Duration:** ________ days ○Unk

☐ **Other**________________________ **Date commenced** [_D_][_D_]/[_M_][_M_]/[_2_][_0_][_Y_][_Y_] ○Unk

**duration:** ________days ○Unk


Adapted from SPRINT SARI CRF by ISARIC. Licensed under a Creative Commons Attribution-ShareAlike 4.0 International License by ISARIC on behalf of Oxford University.

# MODULE 3: OUTCOME CASE REPORT FORM

***MEDICATION (continued):***

**ANTIBIOTIC?** O YES O NO O Unknown **If yes, specify all:**

**Agent 1:**__________________________ **Date commenced** [_D_][_D_]/[_M_][_M_]/[_2_][_0_][_Y_][_Y_] **Duration:** ____ days O Unk

**Agent 2:**__________________________ **Date commenced** [_D_][_D_]/[_M_][_M_]/[_2_][_0_][_Y_][_Y_] **Duration:** ____ days O Unk

**Agent 3:**__________________________ **Date commenced** [_D_][_D_]/[_M_][_M_]/[_2_][_0_][_Y_][_Y_] **Duration:** ____ days O Unk

---

**CORTICOSTEROID?** O YES O NO O Unknown

**If YES:** **Dexamethasone?** O YES O NO O Unknown

**If YES, check all that apply:**

☐ **6mg once per day (od)?** O YES O NO O Unknown If YES, Route: ☐ Oral ☐ Intravenous O Unk

If YES, **Date commenced** [_D_][_D_]/[_M_][_M_]/[_2_][_0_][_Y_][_Y_] **Duration:** ______ days O Unk

☐ **other dose or frequency?** O YES O NO O Unknown If YES, Route: ☐ Oral ☐ Intravenous O Unk

If YES, **Date commenced** [_D_][_D_]/[_M_][_M_]/[_2_][_0_][_Y_][_Y_] **Duration:** ______ days O Unk

**If YES:** **Other corticosteroid?** O YES O NO O Unknown

If YES: **Which steroid:** ☐ Prednisolone ☐ Hydrocortisone ☐ Methylprednisolone ☐ Other

**Route:** ☐ Oral ☐ Intravenous O Unk

---

**ANTICOAGULATION?** O YES O NO O Unk

**If YES: Agent:** ________________________________

**Route:** ☐ Subcutaneous ☐ Intravenous (IV) O Unk

**Indication:** ☐ therapeutic (treatment of DVT/PE) ☐ enhanced prophylaxis for COVID-19 ☐ routine inpatient prophylaxis ☐ Unk

---

**ANTIFUNGAL AGENT?** O YES O NO O Unk

---

**OTHER treatments administered for COVID-19 including experimental or compassionate use?** O YES O NO O Unk

**If YES, specify agent and timing of administration:**

**Agent 1:** __________________________

**Date commenced** [_D_][_D_]/[_M_][_M_]/[_2_][_0_][_Y_][_Y_] O Unk **Duration:** _______ days O Unk

**Agent 2:** __________________________

**Date commenced** [_D_][_D_]/[_M_][_M_]/[_2_][_0_][_Y_][_Y_] O Unk **Duration:** _______ days O Unk

**Agent 3:** __________________________

**Date commenced** [_D_][_D_]/[_M_][_M_]/[_2_][_0_][_Y_][_Y_] O Unk **Duration:** _______ days O Unk


Adapted from SPRINT SARI CRF by ISARIC. Licensed under a Creative Commons Attribution-ShareAlike 4.0 International License by ISARIC on behalf of Oxford University.

# MODULE 3: OUTCOME CASE REPORT FORM

## OUTCOME

**Outcome:** ○Discharged alive ○Hospitalised ○Transfer to other facility ○Death ○Palliative discharge ○Unknown

**Outcome date:** [_D_][_D_]/[_M_][_M_]/[_2_][_0_][_Y_][_Y_] ○Unknown

**If alive at outcome date:**

**Ability to self-care at discharge versus before illness:** ○Same as before illness ○Worse ○Better ○Unknown

**Post-discharge treatment: Oxygen therapy?** ○YES ○NO ○Unknown

**Ongoing health care needs relating to this admission for COVID-19:** ○YES ○NO ○Unknown

**Ongoing health care needs NOT related to COVID episode:** ○YES ○NO ○Unknown

**Medically fit for discharge (COVID-19 resolved) but remains in hospital for other reason (e.g. awaiting suitable care in community, resident in long term health care or mental health facility):** ○YES ○NO ○Unknown


Adapted from SPRINT SARI CRF by ISARIC. Licensed under a Creative Commons Attribution-ShareAlike 4.0 International License by ISARIC on behalf of Oxford University.

**POST PARTUM (within 6 weeks of delivery)?** O YES O NO O Unknown *(if NO or Unknown skip this section)*

**Pregnancy Outcome:** O Live birth O Still birth **Delivery date:** [_D_][_D_]/[_M_][_M_]/[_2_][_0_][_Y_][_Y_]

**Baby tested for COVID-19/SARS-CoV-2 infection?** O YES O NO O Unknown

**If YES, result of test:** O Positive O Negative O Unknown (If Positive, complete a separate CRF for baby)

**INFANT – Less than 1 year old?** O YES O NO *(If NO skip this section)*

**Birth weight:** [___][___].[___] O kg or O lbs O Unknown

**Gestational outcome:** O Term birth (≥37wk GA) O Preterm birth (<37wk GA) O Unknown

**Breastfed?** O YES-currently breastfeeding O YES-breastfeeding discontinued O NO O Unknown

**Vaccinations appropriate for age/country?** O YES O NO O Unknown

## PREVIOUS COVID-19 INFECTIONS

**Has the patient had COVID-19 previously?**

O No O Yes - once previously O Yes - twice previously O Yes - three times previously

(*there is more space on the eCRF to capture this)*

**First COVID-19 infection: When did their first COVID infection occur?** (MM/YYY) ___________

**Was their first COVID infection confirmed by testing:** O Yes, confirmed by testing O No, not confirmed by testing

**Were they admitted to hospital for their first infection of COVID?** O Yes O No

**Second COVID-19 infection: When did their second COVID infection occur?** (MM/YYY) ___________

**Was their second COVID infection confirmed by testing:** O Yes, confirmed by testing O No, not confirmed by testing

**Were they admitted to hospital for their second infection of COVID?** O Yes O No

*If data on this patient was previously recorded in this study, record the Participant Identification Number (PIN) previously used in the section below*

## RE-ADMISSION AND PREVIOUS PIN

**Was the patient admitted previously or transferred from any other facility during this illness episode?**

O YES-admitted previously to this facility O YES–transferred from other facility O NO O Unknown

**Number of previous admissions for this infection:** ______

**Has this patient's data been previously collected under a different patient number?** O YES O NO O Unknown

**If YES, Participant Identification number (PIN):** _________________

## VACCINATIONS

**Covid-19 vaccination:** O YES O NO O Unk

Date of first vaccine :[_D_][_D_]/[_M_][_M_]/[_2_][_0_][_Y_][_Y_] Date: O actual O estimated

Type of first vaccine: O Pfizer/BioNTech | O AstraZeneca Oxford (Covishield in India) | O Moderna | O Novavax
O Janssens (Johnson & Johnson) | O Sinopharm | O Sinovac | O Sputnik V | O Covaxin | O CanSinoBIO
O Unknown | O other, please specify ______________________________

Date of second vaccine :[_D_][_D_]/[_M_][_M_]/[_2_][_0_][_Y_][_Y_] Date: O actual O estimated

Type of second vaccine: O Pfizer/BioNTech | O AstraZeneca/University of Oxford (Covishield in India) | O Moderna | O Novavax
O Janssens (Johnson & Johnson) | O Sinopharm | O Sinovac | O Sputnik V | O Covaxin | O CanSinoBIO
O Unknown | O other, please specify ______________________________

Date of third vaccine :[_D_][_D_]/[_M_][_M_]/[_2_][_0_][_Y_][_Y_] Date: O actual O estimated

Type of third vaccine: O Pfizer/BioNTech | O AstraZeneca/University of Oxford (Covishield in India) | O Moderna | O Novavax
O Janssens (Johnson & Johnson) | O Sinopharm | O Sinovac | O Sputnik V | O Covaxin | O CanSinoBIO
O Unknown | O other, please specify _____________________


Adapted from SPRINT SARI CRF by ISARIC. Licensed under a Creative Commons Attribution-ShareAlike 4.0 International License by ISARIC on behalf of Oxford University.

**Influenza vaccination within the last 6 months:** ○YES ○NO ○Unknown

Date of influenza vaccine :[_D_][_D_]/[_M_][_M_]/[_2_][_0_][_Y_][_Y_] Date: ○actual ○estimated

**Was patient diagnosed with Covid-19? ○YES ○NO ○Unknown**

**If yes, was the diagnosis based on: ○laboratory confirmation ○ clinical assessment**

**Is the patient infected with a variant of concern (VOC)?**

- ○ Unknown
- ○ No: Variant is known and no VOC identified
- ○ Yes: Delta - B.1.617.2, identified Oct 2020
- ○ Yes: Omicron, B.1.1.529, identified Nov 2021
- ○ Yes: Alpha - B.1.1.7, identified in UK Sept 2020
- ○ Yes: Beta - B.1.351, identified in South Africa May 2020
- ○ Yes: Gamma - P.1, identified in Brazil Nov 2020
- ○ Yes: Epsilon - B.1.427/B.1.429, identified in USA Mar 2021
- ○ Yes: Zeta - P.2, identified in Brazil Apr 2020
- ○ Yes: Eta - B.1.525, identified in Multiple Countries Dec 2020
- ○ Yes: Theta - P.3, identified in Philippines Jan 2021
- ○ Yes: Iota - B.1.526, identified in USA Nov 2020
- ○ Yes: Kappa - B.1.617.1, identified in India Oct 2020
- ○ Yes: Lambda - C.37, identified in Peru Dec 2020
- ○ Yes: Mu - B.1.621, identified in Colombia Jan 2021
- ○ Yes: A variant not listed above

*Please check the REDCAP database for variants not listed above. New variants will be added to the database as they are identified.*

**If the Omicron variant was identified, what method was used to identify it?**

○Genomic sequencing ○S-gene target failure (SGTF) testing ○PCR genotyping ○Unknown or untested



Adapted from SPRINT SARI CRF by ISARIC. Licensed under a Creative Commons Attribution-ShareAlike 4.0 International License by ISARIC on behalf of Oxford University.
